# Deoxyuridine in DNA has an inhibitory and promutagenic effect on RNA transcription by diverse RNA polymerases

**DOI:** 10.1093/nar/gkz183

**Published:** 2019-03-20

**Authors:** Junru Cui, Anthony Gizzi, James T Stivers

**Affiliations:** Department of Pharmacology and Molecular Sciences, The Johns Hopkins University School of Medicine, 725 North Wolfe Street, Baltimore, MD 21205-2185, USA

## Abstract

dUTP is a close structural congener of dTTP and can be readily incorporated into DNA opposite to adenine during DNA replication leading to non-mutagenic dU/A base pairs (‘uracilation’). We find that dU/A pairs located within DNA transcriptional templates optimized for either T7 RNA polymerase (T7 RNAP) or human RNA polymerase II (pol II) have inhibitory and mutagenic effects on transcription. The data for T7 RNAP establishes that even a single dU/A pair can inhibit promoter binding and transcription initiation up to 30-fold, and that inhibitory effects on transcription elongation are also possible. Sequencing of the mRNA transcribed from uniformly uracilated DNA templates by T7 RNAP indicated an increased frequency of transversion and insertion mutations compared to all T/A templates. Strong effects of dU/A pairs on cellular transcription activity and fidelity were also observed with RNA pol II using uracil base excision repair (UBER)-deficient human cells. At the highest levels of template uracilation, transcription by RNA pol II was completely blocked. We propose that these effects arise from the decreased thermodynamic stability and increased dynamics of dU/A pairs in DNA. The potential implications of these findings on gene regulation and disease are discussed.

## INTRODUCTION

Deoxyuridine-deoxyadenosine base pairs arise from the incorporation of dUTP during DNA replication because most DNA polymerases are not capable of distinguishing between dUTP and TTP (1). dU/A pairs in DNA are not generally considered as potential modulators of gene expression at the transcriptional level for at least two reasons. First, the abundance of uracil in genomes of dividing cells is generally low due to efficient uracil base excision repair systems (2,3). Secondly, dU/A and T/A base pairs are structurally similar and replicated with high fidelity by most DNA polymerases, suggesting that the activity of RNA polymerases and transcription factors would not be greatly impacted by the presence of dU/A pairs in DNA (4,5).

There are increasing reasons to anticipate that genomic dU/A pairs are present at high densities under certain conditions and that dU/A is not equivalent to T/A in the context of various DNA transactions. The assumption that dU/A pairs are present at vanishingly low levels is being challenged by new detection methods that can map uracil densities in genomic DNA samples (6–8). These methods have revealed genomic hotspots rich in dU/A pairs that could impact protein interactions with DNA. In dividing human cells, regions containing relatively high densities of uracil include telomeres and centromeres (8,9). In the case of telomeres, the presence of dU/A pairs has been shown to impede shelterin binding, leading to defective maintenance of telomere length in B cells (9). High levels of uracil enrichment were also detected in the centromeric region of human chromosomes, especially near the binding site of histone H3-like centromeric protein A (CENP-A) (8). Finally, in developing embryos of drosophila genomic uracil levels in the range 200–2000 uracils/million bases have been detected, while dU/A pairs were found at significantly lower levels in mature flies (10). In flies, DNA uracilation was found to be important for normal metamorphic development and a gradual shift from dU/A → T/A was shown to take place under the spatiotemporal control of two enzymes, dUTPase (which depletes the dUTP pool) and uracil DNA glycosylase (which initiates repair by excising uracil from the DNA backbone) (10–12).

The assumption that dU/A is always equivalent to T/A with respect to protein-DNA interactions is clearly not always justified. For instance, one or two dU/A base pairs within the specific cleavage site of some restriction enzymes can abrogate DNA strand cleavage (13,14). In addition, dU/A pairs have been found to inhibit AP-1 transcription factor binding to DNA (15) and DNA containing a single deoxyuridine residue can disrupt RNase H splicing specificity during *in vitro* reverse transcription (16). Taken in the entirety, the above findings suggest that high local densities of dU/A pairs exist in genomes and have the potential to impact diverse biological functions. However, there has been no investigation exploring whether dU/A pairs might impact gene transcription in human cells.

One emerging area where uracilation can impact human disease is during HIV-1 infection of non-dividing macrophages (6,17,18). In this case, high levels of uracil in viral cDNA occurs as a result of reverse transcription in a cellular environment that contains a highly elevated ratio dUTP/TTP ∼ 20–60 (6,7,18). Because of the low uracil base excision repair (UBER) activity in these cells, many of the viral cDNA products escape uracil excision/DNA fragmentation and integrate into the host cell genome intact (7). The density of dU/A pairs in proviral DNA has not yet been quantified, but a lower limit indicates that every 100 bp segment of the viral LTR promoter region contains more than one uracil on both strands (and likely much higher levels given the high dUTP/TTP ratio) (7). Due to the low UBER activity in these cells, proviral uracils in cultured monocyte-derived macrophages (MDM) can persist for at least 30 days (7). The long-term persistence of uracils in proviral DNA and their possible effects on protein binding and enzyme activity suggests that uracilation could play a significant role in viral latency in macrophages.

In this work we explore the transcriptional and mutagenic effects of dU/A pairs placed at specific sites and increasing densities in DNA templates for transcription. The significant effects of DNA uracilation on the comparatively simple T7 RNAP system as well as the highly complex human RNA pol II establishes that uracil discrimination by RNA polymerases is an evolutionarily conserved property that provides a selective advantage. In the case of HIV infection of macrophages, integrated uracilated proviruses may remain transcriptionally dormant promoting long term latency in this potential viral reservoir.

## EXPERIMENTAL PROCEDURES

### Enzymes and DNA templates

T7 RNAP was purchased from New England Biolabs (NEB, MA, USA). The enzyme concentration was determined based on its specific activity. The linear DNA substrate (S321) containing various levels of dU/A pairs was prepared by PCR amplification in the presence of increasing dUTP:TTP using Taq DNA polymerase (NEB, MA) and pET30a as a DNA template. (S321 primers: Forward; 5′-GAA ATT AAT ACG ACT CAC TAT AGG GGA ATT G-3′. Reverse; 5′-TTT GTT AGC AGC CGG ATC TC-3′). The PCR products were purified using a QIAquick PCR Purification Kit (Qiagen) and quantified by absorbance at 260 nm. The presence of uracil in the templates was confirmed by digestion with uracil DNA glycosylase (UNG), heating for 15 min at 70°C, followed by agarose gel electrophoresis and ethidium bromide staining to confirm DNA fragmentation.

### Oligonucleotides

Oligonucleotides were purchased from Integrated DNA Technologies (IDT; Coralville, IA, USA). The concentration was confirmed from absorbance at 260 nm and the calculated molar extinction coefficients. The purity of the oligonucleotides was confirmed by electrophoresis through a denaturing (8 M urea) 20% polyacrylamide gel. Double-stranded DNA was prepared by heating the complementary single strands to 90°C for 5 min in a buffer containing 40 mM HEPES, pH 7.8 and 1 mM EDTA, then allowing the resulting mixture to cool to room temperature over one hour. In the annealing step, the non-transcribed strand was provided in 10% excess to ensure complete annealing of the template strand. The formation of duplex DNA was confirmed by electrophoresis using a native 20% polyacrylamide gel. The sequences of the oligonucleotides used in this study are listed in the Supporting Information.

### Steady-state kinetics of T7 RNAP transcription

Kinetic assays of *in vitro* transcription on DNA templates with varying uracil content were carried out in 20 μl of reaction solution containing 0.05% (w/v) Tween-20, 175 mM KGlu, 30 mM HEPES (pH 7.8), 15 mM MgSO_4_, 0.25 mM EDTA, 1 mM DTT, 0.5 mM NTP mix and 100 μCi/ml of α-^32^P GTP (Perkin Elmer). Varying concentrations of DNA templates and 5, 10 nM T7 RNAP (New England Biolabs) were used for S321, S23 and S38 respectively. The reactions were incubated at 37°C. Five μl portions of the reaction were removed at 0, 5, and 10 min and mixed with 15 μl of loading buffer (98% formamide, 10 mM EDTA). The transcription products were separated using denaturing PAGE (8 M urea, 1× TBE, 6–14% acrylamide). The gels (20 × 16 cm, 0.3 mm) were pre-run at 25 W for 15 min. Five microliters of each time sample was loaded into a gel well without further treatment. The gels were run for various times to optimize separation of the RNA products. After electrophoresis, ^32^P-labeled RNA products were visualized by exposing the gel to a phosphor screen overnight. The phosphor screens were imaged (Typhoon 9500 imager; GE Healthcare) and the images were quantified using Image Lab software (Bio-Rad). Automatic background subtraction was used and the numbers of counts were normalized to an internal reference of α-^32^P GTP that was spotted on filter paper. The kinetic parameters *k*_cat_ and *K*_m_ were calculated via Prism 7.04 using Equation (1).

(1) \begin{equation*}V\ = {\raise0.7ex\hbox{$1$} \!\mathord{\left/ {\vphantom {1 2}}\right.} \!\lower0.7ex\hbox{$2$}}\ {k_{{\rm cat}}}\left\{ {a - {{\left[ {{a^2} - 4{{\left[ E \right]}_{{\rm tot}}}\left[ {{\rm DNA}} \right]} \right]}^{{\raise0.7ex\hbox{$1$} \!\mathord{\left/ {\vphantom {1 2}}\right.} \!\lower0.7ex\hbox{$2$}}}}} \right\}\end{equation*}

Equation (1) is a variation of the Michaelis–Menten equation that is useful under conditions where the assumption that [ES] << [S] does not hold (19). In this equation, *a* = [DNA] + [E]_tot_ + *K*_m_, *V* is the rate of incorporation of dGMP and [E]_tot_ is the total concentration of T7 RNAP in the reaction.

All initial rate kinetic measurements were performed by taking two time points in the initial rate regime and the amount of full-length transcription product at each time was fit by linear regression to obtain the velocities (slopes). All kinetic experiments were performed at least twice and the replicate measurements of the slopes were plotted against DNA substrate concentration and fit to Equation (1) using non-linear regression analysis in Prism. The reported errors (standard deviations) are the deviation of the data from the fitted theoretical curve.

### Transcriptional error frequency measurements using end-point limiting-dilution clonal sequencing

To determine if DNA template uracils affected the error frequency of T7 RNAP, runoff transcripts were generated using a 1095 bp DNA template that contained all T/A and all dU/A pairs. We then used a limiting-dilution sequencing approach (7,20) to directly sequence cDNAs derived from clonal mRNAs produced from the all T DNA template (T1095) and the same template where all the T/A pairs were dU/A (U1095). To generate these templates, a segment of psiCHECK2 plasmid (666–1761) (Promega) containing the T7 promoter was PCR amplified in the presence of dUTP or TTP (ProFlex PCR System; Applied Biosystems) using Taq polymerase and the following primers: Forward 5′-TAA TAC GAC TCA CTA TAG GCT AGC C-3′, Reverse 5′-GGT CCG AAG ACT CAT TTA GAT CC-3′). The PCR protocol was: initial denaturation at 95°C for 1 min, 35 cycles consisting of 30 s of denaturation at 95°C, 30 s of annealing at 55°C and 2.5 min of extension at 68°C. The final extension was 2 min at 68°C and the samples were held at 4°C until further processing. The resulting all T and all U PCR products were column purified and quantified by UV absorbance at 260 nm. Transcription reactions using T7 RNAP were used to prepare runoff transcription products from the T1095 and U1095 templates. Fifty microliter reactions containing 100 nM T7 RNAP and T1095 or U1095 were performed at 37°C for 2 h using the transcription buffer described above. The reactions were then treated with DNase I (2 units) for 30 min to digest the carryover DNA templates. The RNA transcription products were purified using RNeasy Mini Kit (Qiagen) following the manufacturer's instructions and quantified by UV absorbance at 260 nm. RT-PCR (RT-S1095 primer: 5′-CTT CTT AGC TCC CTC GAC AAT AG-3′) was then used to generate cDNA from equal amounts of each RNA derived from the two DNA templates (High-Capacity cDNA Reverse Transcription Kit; Applied Biosystems). Quantitative real-time PCR with SYBR green detection was used to measure the cDNA copy number resulting from each RT-PCR reaction. Based on the measured copy numbers, dilutions were then designed to give a single copy of cDNA per unit volume. Single copy conditions were assumed when only one of five replicate dilutions were found to be positive by qPCR (∼90% probability of being clonal based on the Poisson distribution). The clonally derived DNA samples were directly sequenced using the Sanger method. Mutations were deemed valid when observed on both sequenced strands using forward and reverse primers (Forward 5′-TAA TAC GAC TCA CTA TAG GCT AGC C-3′, Reverse 5′-GGT CCG AAG ACT CAT TTA GAT CC-3′). Sequences were compared with the original psiCHECK2 plasmid reference sequence using (CLC Sequence Viewer 8, Qiagen). The error frequency was calculated based on the total number of bases sequenced (i.e. error frequency = base error count/total bases sequenced). The same approach was used to determine if DNA template uracils affected the error frequency of human RNA pol II.

### Preparation of uracil containing DNA templates for RNA pol II transcription

Uracilated linear double stranded DNA amplicons containing a canonical CMV promoter coupled to an eGFP gene sequence were prepared by PCR amplification using Taq polymerase and increasing ratios of dUTP/TTP. The template plasmid (pCMV-GFP; ID 11153) was from Addgene. The amplified region of 2344 bp length started ∼550 bp upstream from the CMV promoter/ initiation sequences and ended ∼550 bp downstream of the stop codon for the eGFP gene (Forward primer: 5′-CAG GTC GAG GGA TCT CCA TAA GAG AAG-3′; Reverse primer: 5′-CTG AGA ATA GTG TAT GCG GCG ACC-3′). Each amplicon was generated by performing PCR conducted in a total volume of 500 μl containing 1× ThermoPol Buffer, 10 mM of dATP, dCTP, and dGTP with 10 mM total of dUTP and/or dTTP in noted ratios, 10 μM primers, 5 ng/μl template plasmid, and 5 μl of Taq polymerase. Using this approach, a total of six amplicons (2344 bp) with dU/A contents in the range 5 to 75% were prepared. The presence of uracil was confirmed by treatment with UNG and gel electrophoresis as described above.

### Human cell lines and culturing

HAP1^wt^ and HAP1^ΔUNG^ cell lines were purchased from Horizon Discovery (Cambridge, UK). The HAP1^ΔUNG^ cell line contains a deletion that prevents expression of both the nuclear and mitochondrial forms of uracil DNA-glycosylase (hUNG1 and hUNG2). Both cell lines were cultured with DMEM supplemented with 10% fetal bovine serum (DMEM-10). These lines have been used in our previous studies of the intracellular kinetic properties of hUNG2 (21). Importantly, the HAP1^ΔUNG^ line has been rigorously shown not to possess any intracellular uracil excision activity for substrates containing dU/A pairs (21).

### Transfection of cells

HAP1^wt^ or HAP1^ΔUNG^ cells were plated in T-75 flasks and incubated under standard conditions for 36 to 48 h. The cells were released by treatment with 0.5% Trypsin–EDTA, washed twice with medium and plated in 24-well plates at a density of 0.5 × 10^5^ cells per well. The cells were grown to ∼90% confluence before transfection. Three hours before transfection, the media was replaced with 400 μl of fresh DMEM-10. In a total solution containing 100 μl Opti-MEM medium, 1 μg of each amplicon was mixed with 3 μl of lipofectamine 3000 (Thermo Fisher) and incubated at room temperature for 30 min. The 24-well plate containing either HAP1^wt^ or HAP1^ΔUNG^ cells was removed from the incubator and the 100 μl lipofectamine /DNA solution was applied to each well and mixed by gentle rocking. The transfection cultures were incubated for 24 h, after which the cells were washed with PBS and released by treatment with 0.5% Trypsin-EDTA. The cells were then pelleted by centrifugation at 500 x g for 5 min. The samples were used for either flow cytometry, qPCR quantification of eGFP DNA, or quantification of eGFP mRNA by RT-qPCR.

### Flow cytometry

Following transfection and pelleting, cells were washed twice with PBS, gently resuspended in PBS and then transferred into 5 ml round bottom tubes with a Cell-Strainer Cap (Corning Science). The percent of HAP1^wt^ and HAP1^ΔUNG^ cells expressing eGFP was determined using a Becton Dickinson FACScalibur flow cytometer. A typical sample contained ∼2 × 10^5^ cells in 1 ml of phosphate buffered saline (PBS), and 3 × 10^4^ cells were injected in each flow experiment to obtain good population statistics. The relative percentage of eGFP positive cells is defined as (% positive cells for all U template transfection)/(% positive cells for all T template transfection).

### Quantifying template DNA levels in transfected cells

Following transfection and pelleting, the cells were treated with turbo DNase (ThermoFisher) to remove any extracellular DNA that may be present (10 units, for 30 min at 37°C followed by an additional 10 units and incubation for 30 min). A Qiagen DNA Isolation kit was used to isolate total cellular DNA and the amount of recovered DNA was quantified using a NanoDrop 2000/c. To determine the amount of eGFP present in each sample we used quantitative PCR (qPCR) with primers and probe directed at the eGFP gene (F: 5′- AGA AGT CGT GCT GCT TCA T-3′, R: 5′- GCT GAC CCT GAA GTT CAT CT-3′, Probe: 5′-/56-FAM/AAG CAC TGC/ ZEN/ ACG CCG TAG GT/3IABkFQ/-3′) and the genomic reference gene RPP30 (F: 5′ GAT TTG GAC CTG CGA GCG-3′, R: 5′-GCG GCT GTC TCC ACA AGT-3′, Probe: 5′-HEX-CTG ACC TGA AGG CTC T-MGBNFQ-3′). A total of 50 ng of extracted DNA was used in each qPCR reaction. A Qiagen Rotor-Gene qPCR was used and reactions contained the standard Rotor-Gene PCR mix and the primer/probes at concentrations of 0.4 and 0.2 μM, respectively. The relative copy numbers for the uracilated and all T transfected DNA were calculated from the measured C_t_ values and then normalized to the averaged C_t_ values for the RPP30 genomic reference gene across all the samples (the C_t_ values for the reference gene in each sample were the same within errors of the measurements) (Equation 2):

(2) \begin{equation*}{\rm{Relative}}\,{\rm{copy}}\,{\rm{number}} = {2^{ - (\Delta \Delta {\rm{Ct}})}}\end{equation*}

In Equation (2), $\Delta \Delta {\rm{Ct}} = \frac{{( {{{\rm{C}}_t}^U - {{\rm{C}}_t}^T} )}}{{( {{{\rm{C}}_t}^{RPP30U} - {{\rm{C}}_t}^{RPP30T}} )}}$, where C_t_ values are for the uracil containing DNA (C_t_*^U^*), all T DNA (C_t_*^T^*), or the RPP30 genomic reference for each transfection (C_t_^RPP30^*^U^* and C_t_^RPP30^*^T^*).

### Fraction of transfected DNA that retained uracil

To establish that uracilated DNA recovered from HAP1^ΔUNG^ cells still contained the original levels of uracil, we performed uracil excision qPCR (Ex-qPCR) (17). This method uses UNG treatment of a DNA sample prior to qPCR to determine the fraction of the molecules that contain uracil in the PCR amplicon (i.e. with UNG pretreatment, uracilated DNA copies are selectively removed and subsequent qPCR amplification provides the number of uracil-free copies). In this experiment, 50 ng of extracted DNA from each of the transfected samples was divided in two portions (25 ng each). One portion was treated with bacterial UNG (New England Biolabs) and the second was not. Both portions were analyzed for eGFP copy number using the same qPCR protocol described above. The difference in the C_t_ values for the UNG treated and untreated samples divided by the C_t_ value for the untreated sample is equal to fraction of the DNA that contained uracil in the amplicon (Equation 3), where ΔΔC_t_ = (C_t_^+UNG^ - C_t_^no UNG^)/C_t_^no UNG^.

(3) \begin{equation*}{\rm{Fraction}}\,{\rm{amplicons}}\,{\rm{containing}}\,{\rm{uracil}} = {2^{ - \Delta \Delta {\rm{Ct}}}}\end{equation*}

### Quantifying eGFP mRNA levels in transfected cells using RT-qPCR

Following transfection and harvesting the cell pellets, a Qiagen RNeasy mini kit was used to isolate total cellular RNA from each transfected cell. The amount of recovered RNA was quantified using UV absorbance at 260 nm. To determine the amount of eGFP mRNA present in each sample, we used a random primer reverse transcription method (High-Capacity cDNA Reverse Transcription kit; Applied Biosystems). A total of 50 ng of transcribed cDNA was used for qPCR amplification using a Qiagen Rotor-Gene SYBR green PCR kit. The eGFP copy number was determined relative to a GAPDH reference transcript using specific primers directed at the eGFP gene (F: 5′- AGA AGT CGT GCT GCT TCA T-3′, R: 5′- GCT GAC CCT GAA GTT CAT CT-3′), or GAPDH gene (F: 5′- CGT CTT CAC CAC CAT GGA GAA-3′, R: 5′- ATG GTT CAC ACC CAT GAC GAA-3′). The difference in expression of eGFP mRNA derived from the uracilated DNA templates as compared to the all T template was normalized to the averaged GAPDH expression across all of the samples to calculate the relative eGFP mRNA expression arising from transcription from the uracilated and all T templates using Equation (2), where $\Delta \Delta {\rm{Ct}} = \frac{{( {{{\rm{C}}_t}^U - {{\rm{C}}_t}^T} )}}{{( {{{\rm{C}}_t}^{GAPDH( U )} - {{\rm{C}}_t}^{GAPDH( T )}} )}}$ In this experiment, the C_t_ values for the GAPDH reference in each sample were the same within errors of the measurements

## RESULTS

### Transcription by T7 RNAP is inhibited by dU/A pairs within a 321 bp DNA template

**Figure 1.**
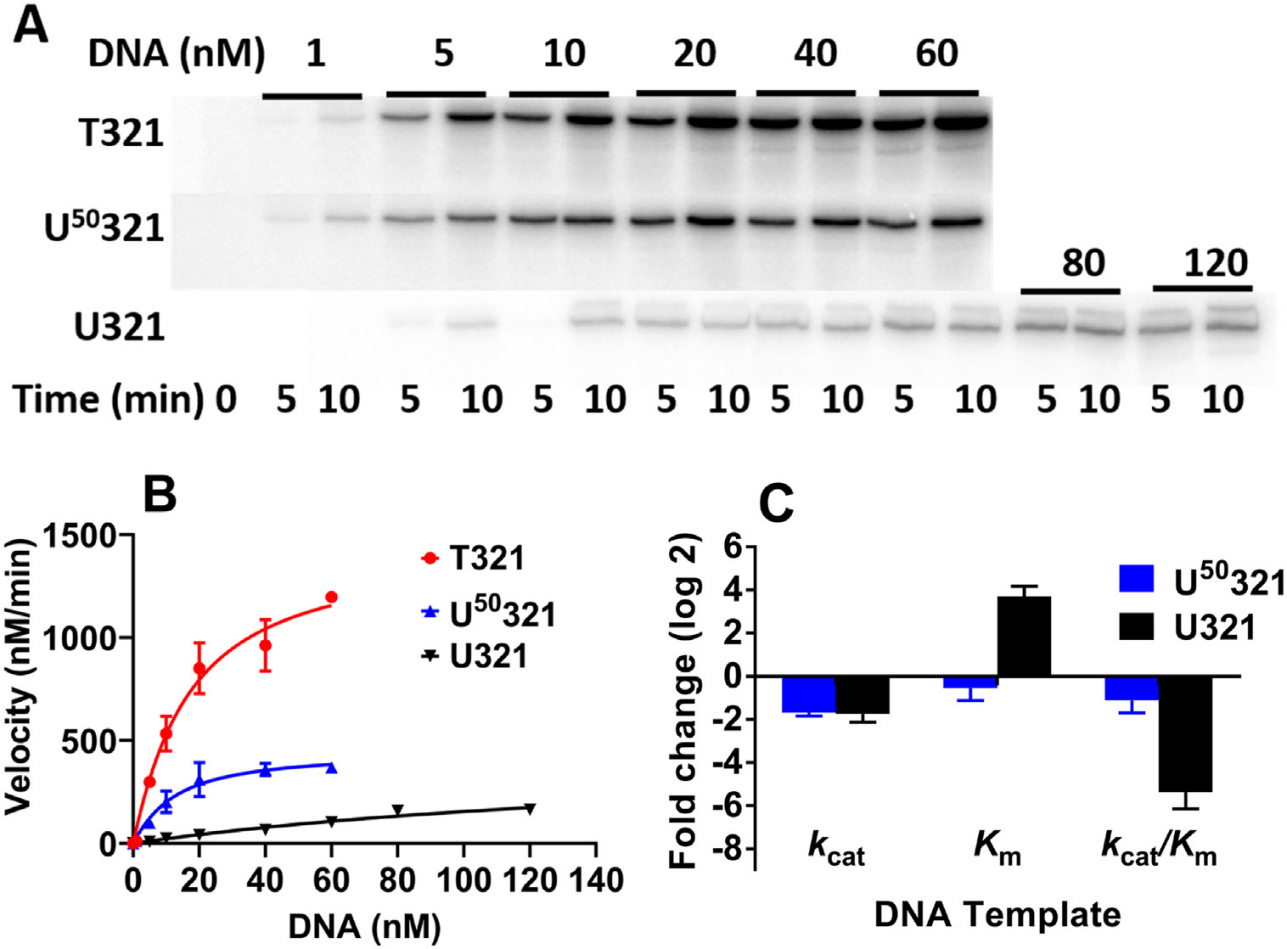
Effect of random T/A→dU/A substitutions on transcription by T7 RNAP using a 321 bp DNA transcription template (S321). (**A**) Representative time courses for transcription of S321 with all T/A and increasing levels of dU/A pairs. (**B**) Kinetics of transcription of S321 containing increasing levels of random dU/A pairs. The curves are non-linear least squares best fits to Equation (1). The reaction conditions were: [DNA] = 1–60 nM (T321 and U^50^321) and 5–120 nM for U321, [T7 RNAP] = 5 nM, [NTP] = 0.5 mM and 100 μCi/ml α-^32^P-GTP. Each measurement was repeated at least twice and the data points are the mean values with indicated standard deviations. For clarity of presentation, only a few representative error bars are shown. The average error over all the measurements was ±18%. (**C**) Comparison of the relative kinetic parameters of S321 with increasing levels of random dU/A pairs. Each parameter for U^50^321 and U321 was normalized to that of T321.

We first sought to understand whether dU/A pairs randomly scattered within a fairly long DNA template could inhibit transcription by T7 RNAP. For *in vitro* transcription assays we used a 321 bp DNA transcription template (S321) containing the highly active T7*lac* promoter found in many pET plasmids used for protein overexpression. Because previously reported *K*_m_ values for T7 RNAP are in the low nanomolar range (22,23), we chose a higher salt concentration (175 mM KGlu) so that more accurate *K*_m_ values could be measured (24). We constructed three DNA templates that contained 0, 50 and 100% T/A→dU/A substitutions by varying the ratio of dUTP:TTP in a PCR reaction with Taq DNA polymerase (T321, U^50^321, and U321, respectively). This substrate contains 84 T/A pairs that will be randomly substituted with U with probabilities of 50 and 100% depending on the dUTP/TTP. Reaction velocities were determined by measuring the steady-state rate of incorporation of α^32^P-GTP into the mRNA transcripts, which were resolved by denaturing (8 M urea) polyacrylamide gel electrophoresis (Figure 1A). Clear inhibitory effects were measured, which could arise from having uracils in either the template or non-template strand, and these effects increased markedly as the density of dU/A pairs increased (Figure 1B, Supplemental Table S1). Analysis of the kinetic data established that the effects on the observed rate arose from changes in *k*_cat_ and/or *K*_m_ (Figure 1C). The maximal effect on the catalytic efficiency (*k*_cat_/*K*_m_) was over 40-fold for U321. These findings indicate that dU/A pairs can decrease transcription by a binding effect (reflected in an increased *K*_m_ value) and to a lesser extent, a decrease in the catalytic activity of the ES complex (*k*_cat_ effects). Since the dU/A pairs are randomly dispersed in the DNA substrate, it is not possible to discern from this data alone whether the effects arise at the stage of promoter binding, transcription initiation or elongation. Further, the data do not reveal whether specific sites of T/A→dU/A substitution are especially important for the observed effects.

### Transcriptional effects of site-specific dU/A pairs within the T7 promoter and initiator regions

The above findings indicate that randomly incorporated dU/A pairs within the entire length of a DNA substrate for transcription lead to a global inhibitory effect on transcription. It is of interest to determine if there are specific hot-spot dU/A pairs in the T7 promoter or transcription initiation regions that give rise to the inhibitory effects.

**Figure 2.**
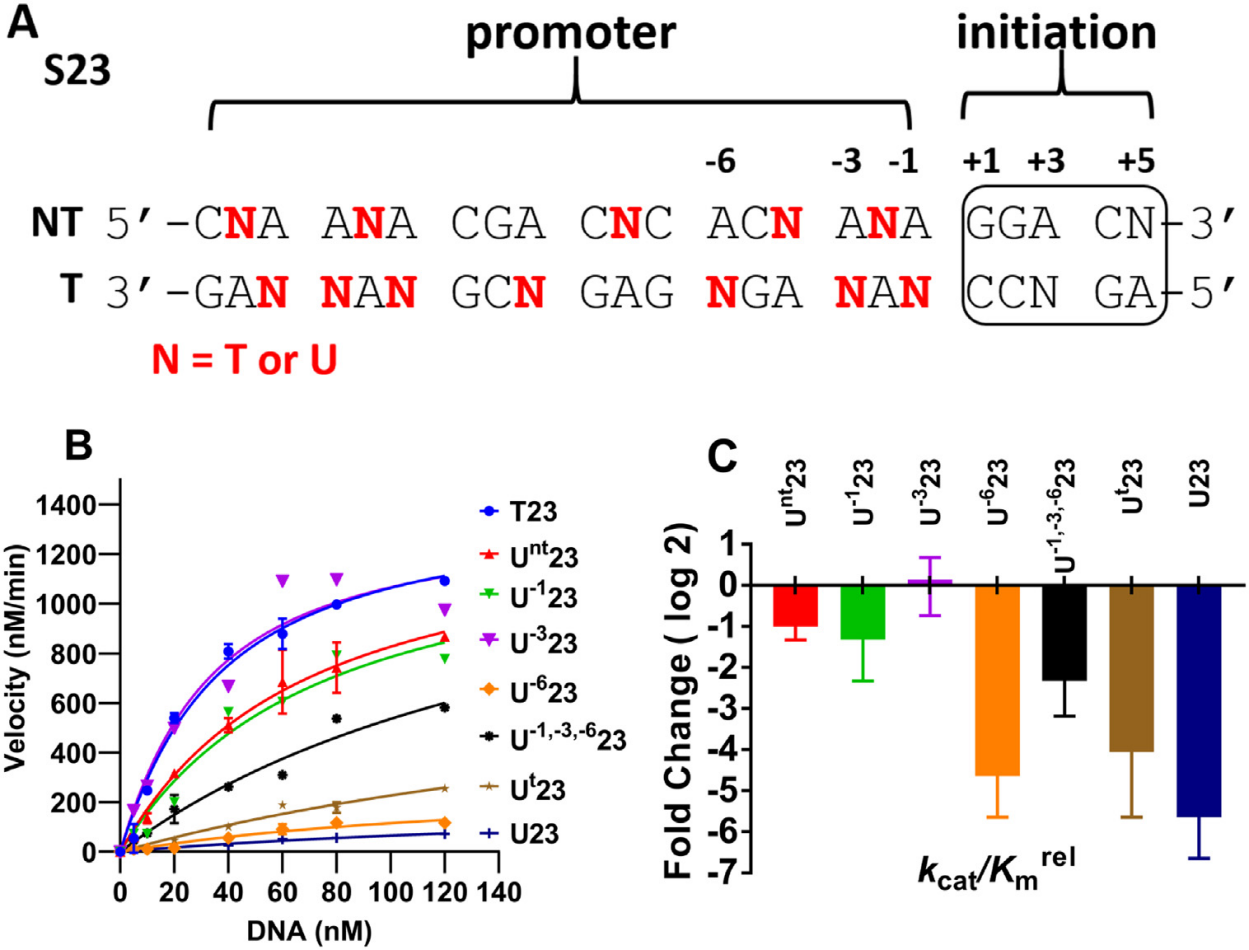
Transcriptional effects of specific dU/A pairs located in a 23 bp DNA (S23) containing the T7 RNAP promoter and initiation sequences. (**A**) S23 sequences with dU/A pairs at the indicated sites (N = T or dU). The boxed sequence is the initiation region (+1 to +5) which generates a short 5 nucleotide transcript. NT and T refer to non-template and template respectively. (**B**) Kinetics of transcription of S23 containing T/A and dU/A pairs at specific sites. The reaction conditions were: [DNA] = 120 nM, [T7 RNAP] = 10 nM, [NTP] = 0.5 mM and 100 μCi/ml α-^32^P-GTP. The curves are non-linear least-squares best fits to Equation (1). The data points are the mean velocities obtained from linear plots of [P] versus time and the indicated errors are standard deviations (*n* = 2–4). For clarity of presentation, only a few representative error bars are shown. The average deviation in replicate measurements was ± 13%. (**C**) Comparison of catalytic efficiencies of T7 RNAP with the indicated substrates. The relative catalytic efficiency (*k*_cat_/*K*_m_^rel^) is the ratio of *k*_cat_/*K*_m_ for the dU/A substrate to that of T23.

To address this question systematically, we synthesized a set of 23 base pair duplexes containing a canonical T7 promoter and initiation sequence with one or more T/A→dU/A substitutions at specific sites and then measured the rate of transcription in the presence of saturating concentrations of NTPs (Figure 2A, Supplemental Table S2). Transcription from this substrate produces a short 5 mer RNA derived from the template strand in the initiation region but does not involve elongation. The major trends in these data are as follows. When uracil was present at every T position on both DNA strands (U23), a 7-fold decrease in *k*_cat_ and a ∼7-fold increase in *K*_m_ were observed, indicating that global uracilation reduces polymerase activity under conditions where the enzyme is saturated with substrate and also at the DNA binding step. When uniform T→dU substitutions were made selectively on the template (t) strand (U^t^23), a ∼2-fold decrease in *k*_cat_ and 8-fold increase in *K*_m_ were observed (Figure 2B, Supplemental Table S2). The similar decrements in *k*_cat_ and *K*_m_ when dU is located on the template strand rather than both stands of the DNA, indicates that the effects are largely derived from disruption of favorable template strand interactions with the polymerase. Consistent with this conclusion, uracil substitutions on the non-template (nt) strand (U^nt^23), resulted in only a small increase in *K*_m_ (∼2-fold), with no change in *k*_cat_ (Figure 2B, Supplemental Table S2).

To localize the effects even further, DNA substrates were investigated that contained single uracil substitutions on the template strand at the -1, -3 or –6 positions of the promoter region of the DNA (U^−1^23, U^−3^23, U^−6^23) (Figure 2A). At the -1 and -3 positions, substitution resulted in only small effects on the kinetic parameters (Figure 2B, Supplemental Table S2). In contrast, a single U^−6^ substitution increased *K*_m_ by ∼4-fold and decreased *k*_cat_ by ∼4-fold. The sensitivity of the –6 position to various base and sugar substitutions has been previously reported (22,25), and establishes that even a single uracil in a promoter region of a gene can dramatically impact gene expression. Although the single -6 substitution recapitulated most of the activity loss that was observed with the uniformly uracilated substrates, the results with the substrate template U^−1,-3,-6^23 revealed energetic antagonism between U^−6^ and the U^−1^ and U^−3^ positions. The antagonism was manifested as a 4-fold rescue of *k*_cat_/*K*_m_ as compared to U^−6^23 (Figure 2C). The fold changes in the *k*_cat_/*K*_m_ values for the eight uracilated S23 DNA substrates as compared to the all T substrate (T23) are summarized in Figure 2C.

**Figure 3.**
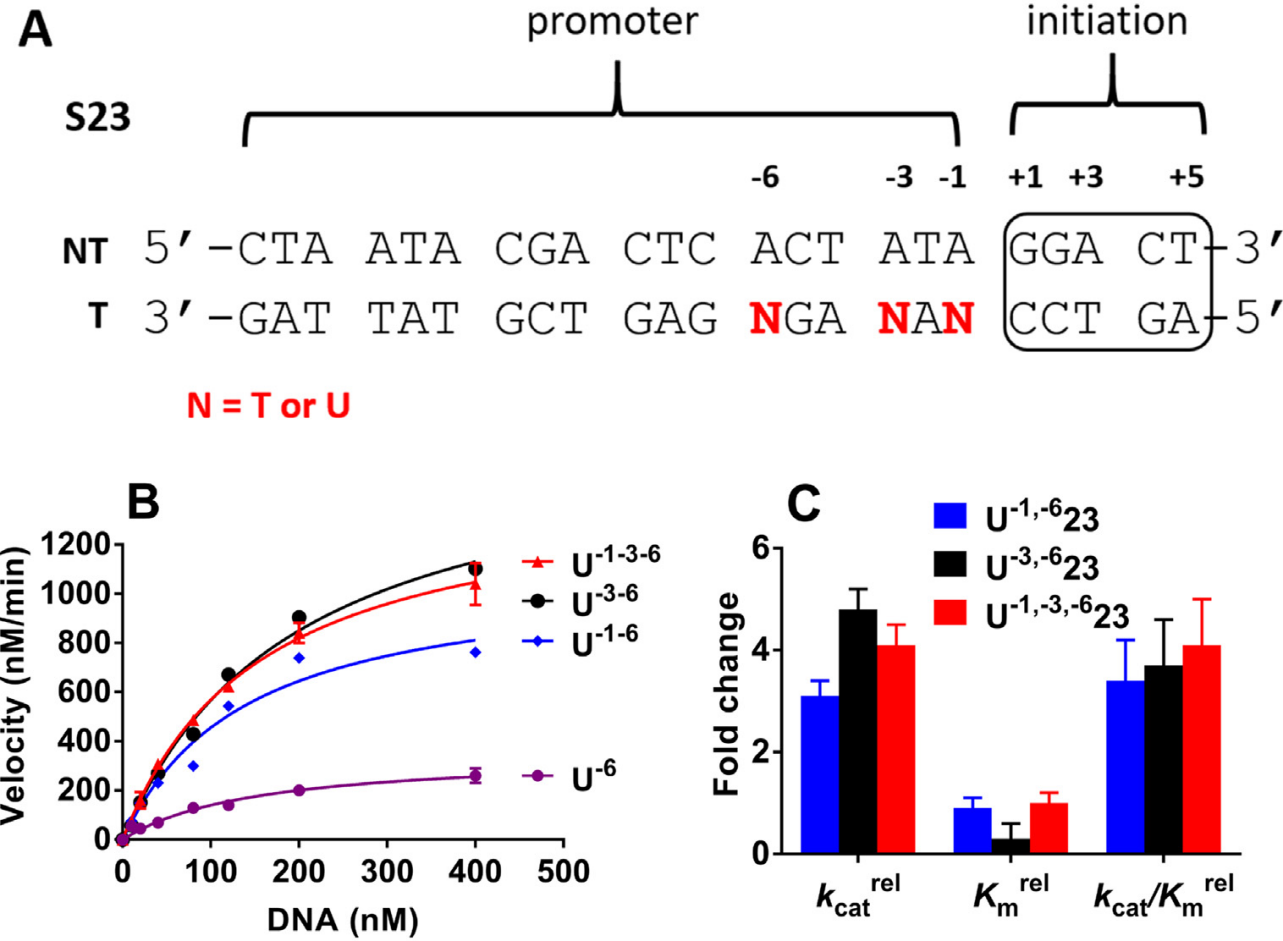
Combinatorial dU/A substitutions at the –1 and –3 positions of S23 rescue the damaging effect of U^−6^. (**A**) Sequences of S23 with uracils at the –6 position in combination with substitutions at the –1, and –3 position (N = T or dU). NT and T refer to non-template and template respectively. (**B**) The inhibitory effect of U^−6^23 is rescued by substitutions at U^−3^ and U^−1^. The reaction conditions were: [DNA] = 120 nM, [T7 RNAP] = 10 nM, [NTP] = 0.5 mM and 100 μCi/ml α-^32^P-GTP. The curves are non-linear least-squares best fits to Equation (1). Each measurement was repeated at least twice and the data points are the mean values with indicated standard deviations. For clarity of presentation, only a few representative error bars are shown. The average deviation in replicate measurements was ±8%. (**C**) Fold increases in the kinetic parameters for U^−1,-6^23, U^−3,-6^23 and U^−1,-3,-6^23 relative to U^−6^23.

We then asked whether single uracil substitutions at the –1 and –3 positions could also rescue the low activity arising from the U^−6^ substitution (Figure 3A). Thus, substrates U^−1,-6^23 and U^−3,-6^23 were synthesized and tested in transcription assays in parallel with U^−6^23 and U^−1,-3,-6^23 (Figure 3B). When combined individually with the U^−6^ substitution, both the U^−1^ and U^−3^ substitutions were also found to rescue the activity loss observed with the U^−6^ substrate to a similar extent to the combined U^−1^ and U^−3^ substitutions (Figure 3C). As compared to *k*_cat_/*K*_m_ of U^−6^23, the U^−1,-6^, U^−3,-6^ constructs enhanced activity by three to five-fold. The *k*_cat_/*K*_m_ effects were derived mainly from changes in *k*_cat_ (Figure 3C).

### Effect of dU/A substitutions during transcription elongation

**Figure 4.**
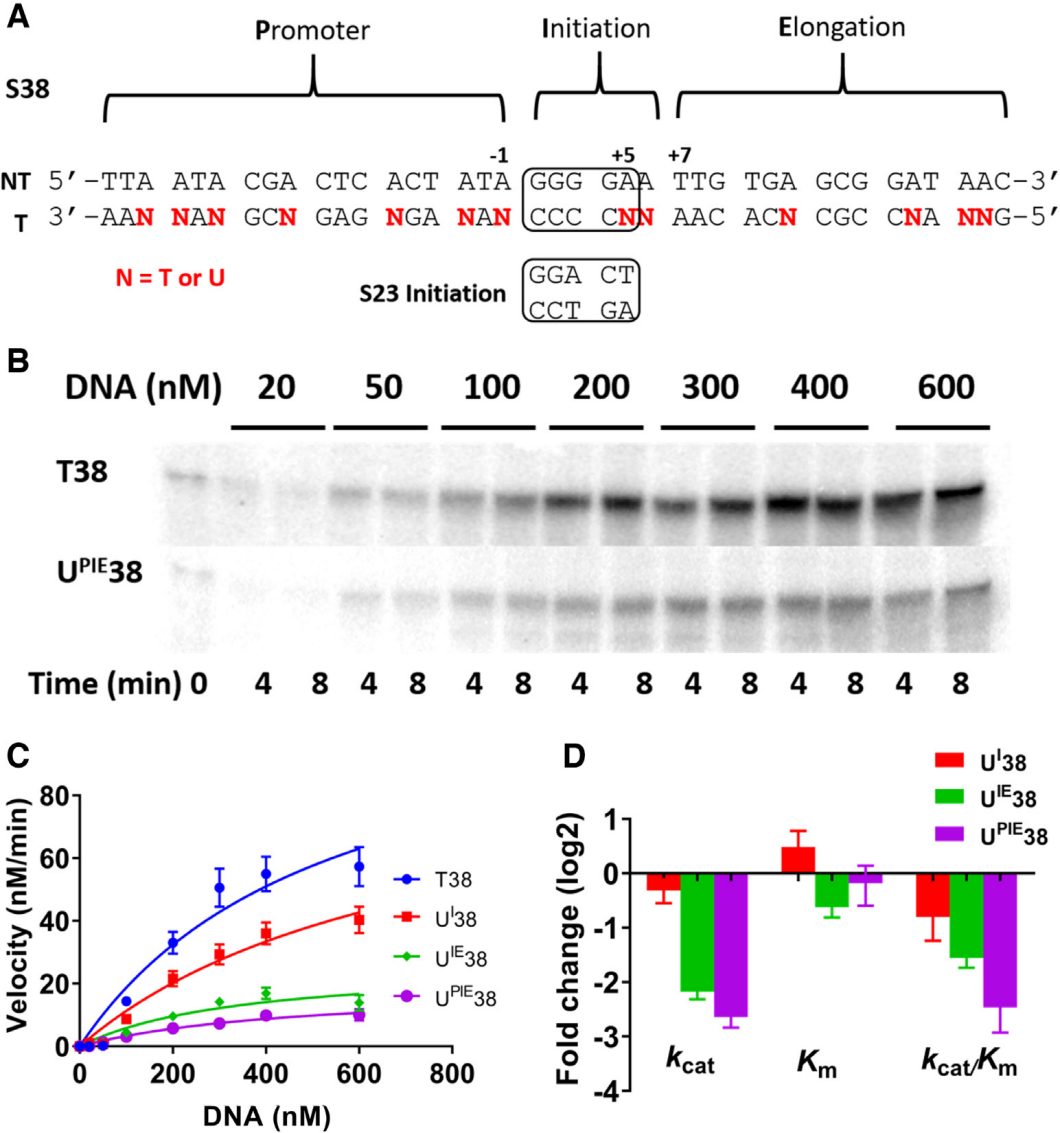
Transcriptional effects of dU/A pairs located in the DNA promoter (P), initiation (I) and elongation (E) sequences for T7 RNAP. (**A**). Sequences of S38 PIE substrate with dU/A pairs in either the I, IE or PIE regions (N = T or dU). NT and T refer to non-template and template respectively. (**B**). Time course for transcription of T38 and U^PIE^38. **(C)** Concentration dependences of the rate of transcription for the four S38 substrates. The reaction conditions were: [DNA] = 20–600 nM, [T7 RNAP] = 20 nM, [NTP] = 0.5 mM and 100 μCi/ml α-^32^P-GTP. The curves are non-linear least-squares best fits to Equation (1). Each measurement was repeated at least twice and the data points are the mean values with indicated standard deviations. For clarity of presentation, only a few representative error bars are shown. The average error over all the measurements was ±11%. (**D**). Fold changes in the kinetic parameters for U^I^38, U^IE^38, and U^PIE^38 relative to T38.

The above experiments revealed that (i) dU/A pairs have global inhibitory effects on transcription when a longer 321 bp transcription template is used, and (ii) a single dU/A pair at a critical site (–6) can inhibit transcription from a short template containing the promoter and transcription initiation elements. To address whether dU/A pairs impacted the elongation stage of transcription that is not present with S23 transcription, we designed a 38 bp substrate that contained the T7 **p**romoter (**P**) and **i**nitiation (**I**) sequence and also a 15 bp region for **e**longation (**E**) (‘PIE’ substrate, S38). The initiation sequence in S38 matches the highly active promoter in S321 but differs from the canonical T7 sequence in S23 (Figure 4A). Uracil substitutions were made on the template strand in the **I, IE** and **PIE** regions of S38 (U^I^38, U^IE^38 and U^PIE^38) and the kinetic behavior was compared with the corresponding all T DNA (T38) (Figure 4B–D). The relative rates of GTP incorporation for these four templates followed the trend T38 > U^I^38 > U^IE^38 > U^PIE^38 (Figure 4C, D). For the S38 series, the kinetic effects were mostly derived from decreases in *k*_cat_ that were as large as 6-fold for U^PIE^38 (Figure 4D, Supplemental Table S3). Of interest, the template U^IE^38 has a much-reduced transcription rate compared to U^I^38. The only difference between these two templates is the presence of uracils in the elongation (**E**) region of the template strand of U^IE^38. This result indicates that dU/A pairs can impact elongation in addition to the previous observed effects on promoter binding and initiation. The template that contains uracil substitutions in all three regions (U^PIE^38) shows an even stronger inhibitory effect, but the addition of promoter uracils is less damaging in S38 than S23. The lesser effect of uracil substitution in the promoter region of S38 as compared to S23 is likely related to the different sequences in the initiation regions of these two templates (Figure 4A). The optimized promoter-initiation sequence of S38 (which is contained in all plasmid-based T7/*lac* expression systems) could increase the efficiency of moving from the initiation to elongation step of transcription. In addition, the kinetic effects arising from uracil substitutions in each substrate would be expected to differ because S38 can elongate after initiation. Thus, the steady-state rates for S23 and S38 are weighted towards different steps of the reaction. Nevertheless, the increased damaging effect of substituting uracils in the **E** region of S38 as compared to **I** alone indicates that dU/A pairs can impact elongation in addition to promoter binding and initiation.

### Transcription fidelity of T7 RNAP with uracilated DNA templates

The fidelity of RNA transcription by single-subunit RNA polymerases such as T7 RNAP is remarkably high (average base substitution frequency ∼10^−6^) (26), while multi-subunit RNA polymerases such as nuclear pol II are show lower fidelity in various assays (10^−3^ to 10^−5^) (27–30). Such transcriptional errors can disrupt many biological processes including RNA splicing, reverse transcription, and mRNA translation leading to disease (31–33). The error frequencies for T7 RNAP have been measured with templates containing T/A pairs, but the possible transcriptional errors resulting from a uracil base in the DNA template has never been explored to our knowledge. To investigate this question, we performed end-point limiting-dilution PCR (EPLD-PCR) to obtain cDNAs from single RNA transcripts of T7 RNAP followed by direct Sanger sequencing. PCR amplification of single molecules after LD circumvents many of the shortfalls of PCR applied to mixtures of sequence variants such as template jumping, allelic preference or biased amplification of one sequence during early PCR cycles (34,35). The LD-sequencing approach also minimized polymerase errors because PCR amplification begins from a single cDNA clone and any errors detected in the Sanger sequencing reads have to occur in the first PCR amplification cycle, which is a low probability event (35).

Nineteen cDNA clones obtained from reverse transcription of the RNA products of T7 RNAP transcription of the T1095 DNA (T/A) template were sequenced and compared with the reference plasmid sequence (Table 1). All sequence electropherograms for each clone obtained by EPLD-PCR contained no double peaks indicative of a mixture containing more than one sequence, and no mutations were observed in transcripts derived from the T/A template after sequencing a total of ∼18 000 bp, which is consistent with the previously reported error rate of T7 RNAP. The upper limit mutation frequency calculated from this result (<6 × 10^−5^/bp) also sets the upper limit for the combined frequency of introducing mutations in the sequenced DNA by any polymerase step that was employed in the DNA template synthesis and RT-PCR processes. Consistent with this upper limit, the error frequency of the Taq DNA polymerase used for DNA template synthesis is 4 × 10^−5^/bp and M-MLV reverse transcriptase used for RT-PCR is 3.3 × 10^−5^/bp (36,37).

**Table 1.** T7 RNAP transcription error frequencies arising from DNA templates containing T/A or dU/A pairs

				Transcriptional mutations			
Template^a^	Clones sequenced^b^	Bases sequenced^c^	Mutated clones	substitution	deletion	insertion	Error frequency^d^ (×10^−4^)
T1095	19	18 014	0	0	0	0	ND
U1095	17	14 616	2	1 (C→A)	0	1 (U)	1.4

^a^The DNA templates T1095 (T/A) and U1095 (dU/A) were obtained by PCR amplification using Taq DNA polymerase (see methods).

^b^Clonal DNA was obtained by limiting-dilution PCR. Single molecule conditions were assumed when only one of five replicate dilutions gave a positive PCR signal (∼90% probability of being clonal).

^c^Total base pairs sequenced is the sum over all clones sequenced.

^d^Mutation frequency = total mutations/total bases sequenced. The reference error frequency of T7 RNAP and M-MLV reverse transcriptase are 2 × 10^−6^ and 3.3 × 10^−5^, respectively (26,36). ND; no errors detected.

**Figure 5.**
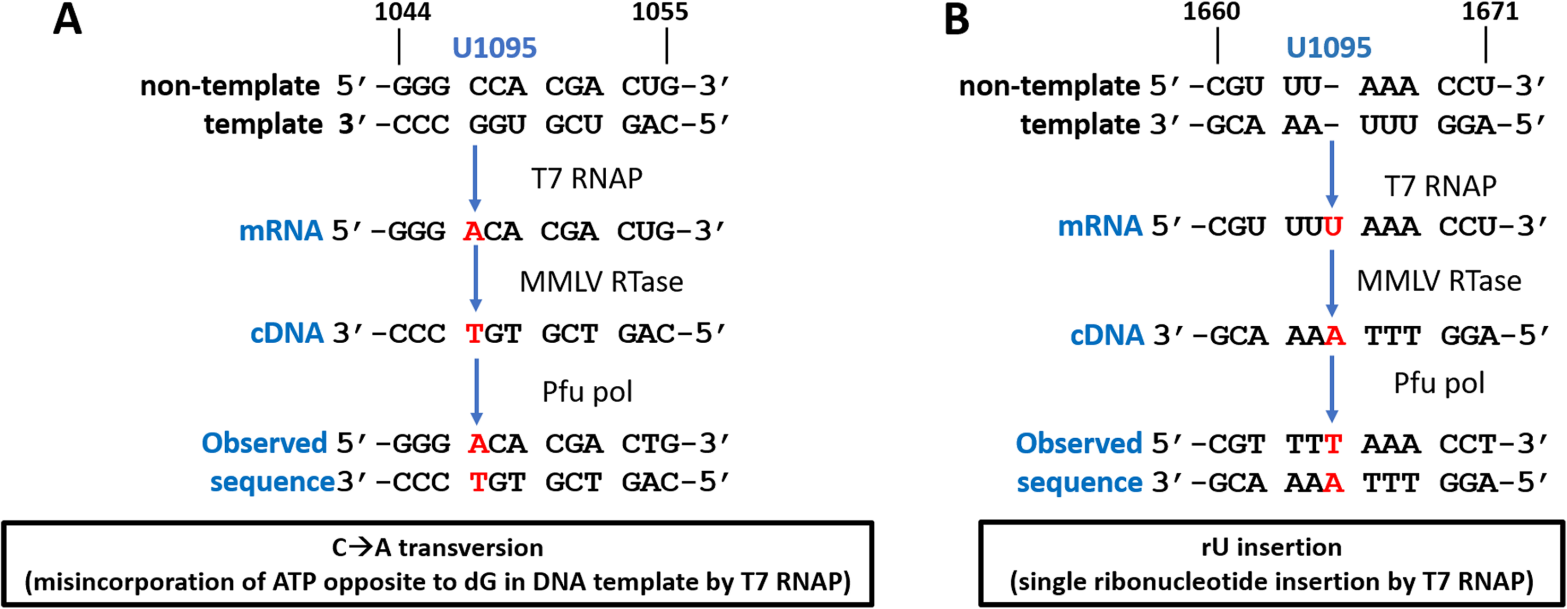
Transcriptional mutations observed with T7 RNAP transcription from the uniformly uracilated DNA template. (**A**) Sequence context of the C→A transversion. Red letters refer to the position of the substitution and the numbers on the top of the sequence correspond to the positions on the original psiCHECK2 plasmid sequence. (**B**) Sequence context analysis of the U insertion mutation in the homopolymer run AAA on the template strand. These mutations were observed on both strands of the Sanger sequencing reads.

Seventeen cDNA clones were obtained from T7 RNAP transcription of the U1095 DNA (dU/A) template and two clonal mutations were observed after sequencing a total of ∼15 000 bp, (Table 1, Supplemental Figure S1). One mutation was a C→A base substitution corresponding to position 1047 of the reference DNA, indicating that ATP mispaired with dG on the DNA template during T7 RNAP transcription (see Discussion) (Figure 5A). The second mutation was an insertion of a U in the mRNA between positions 1664 and 1666 in the reference DNA sequence (Figure 5B). The sequence context for this insertion falls in a homopolymeric run of adenines in the DNA template, which is known to promote strand slippage by polymerases and can lead to insertions or deletions depending on whether the slippage occurs on the extending strand or the template (see Discussion). We note that neither of these mutations occurs at a position corresponding to a uracil base on the DNA template and suggests that the effects of dU/A pairs are mediated through a perturbation of the DNA local structure or stability. In addition, we detected no sequence changes in uracilated DNA templates that were prepared by *in vitro* polymerization in the presence of dUTP (see Methods). This establishes that the observed mutations in the transcripts derived from the uracilated DNA template do not arise from sequence changes in the DNA template. The possible mechanisms for these observed mutations are explored further in the Discussion.

### Effect of dU/A pairs on transcription from a CMV promoter in human cells

We next moved to a human cell model to explore whether dU/A pairs within a CMV promoter could also inhibit transcription by human RNA pol II. Linear dsDNA amplicons with different densities of dU/A pairs were obtained by PCR amplification in the presence of increasing ratios of dUTP:TTP using a 4479 bp plasmid template sequence that contained a CMV promoter coupled to an eGFP gene. The resulting linear amplicons (∼2500 bp) contained between 5% and 75% uracil on both DNA strands in the form of dU/A pairs. The presence of uracil was confirmed by digestion with uracil DNA glycosylase and heating to promote strand breakage at abasic sites (Supplemental Figure S2). The uracilated constructs were used to transfect either wild-type HAP1 cells, (HAP1^wt^), or HAP1^ΔUNG^ which has a deletion in the gene that codes for both nuclear and mitochondrial uracil DNA glycosylase (hUNG). This cell line has no detectable expression of hUNG and no uracil excision activity using DNA substrates with dU/A pairs (21). Accordingly, transfection of this cell line with the uracilated reporter DNA allows assessment of whether intact dU/A pairs have a negative effect on transcription by RNA pol II in the absence of uracil excision or DNA repair. In contrast, in the wild-type cell line the heavily uracilated DNA is expected to be fragmented by the combined action of hUNG and APE1 endonuclease, resulting in a large deleterious effect on mRNA production and eGFP expression.

**Figure 6.**
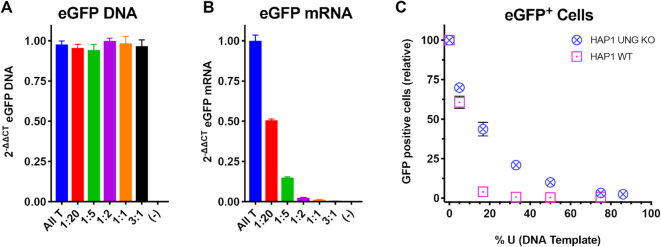
Effects of template dUMP on transcription and protein expression in human cells. (**A**) DNA constructs containing a CMV promoter to drive an eGFP expression cassette and increasing levels of dU/A pairs were transfected into the human HAP1^ΔUNG^ cell line that has no uracil excision activity. The relative copy number of the intracellular transfected DNA was determined by qPCR 24h after transfection. ΔΔC_t_ is defined in the Methods and quantifies the efficiency of each transfection relative to the all T DNA and normalizing to the genomic copies of the RPP30 gene. No transfection control is denoted as (–) (**B**) The mRNA transcripts produced 24 h after transfection with the uracilated DNA templates were quantified using RT-qPCR. ΔΔCt is defined in the Methods and quantifies the mRNA produced relative to the all T DNA, while normalizing to cellular GAPDH expression. No transfection control is denoted as (–) (**C**) The fraction of eGFP positive cells 24 h after transfection was determined by flow cytometry. Transfections and flow cytometry were performed on HAP1^wt^ or HAP1^ΔUNG^ cells and the relative eGFP expression levels were normalized to the number of GFP positive cells obtained with the all T DNA.

Following transfection with the all T and five different uracilated DNA amplicons for 24 h, total cellular DNA was extracted from the HAP1^ΔUNG^ cells. To establish that each uracilated DNA construct was present at the same level in cells as the all T reference DNA, quantitative PCR (qPCR) was performed on DNA isolated from the transfected cells. The copy number of eGFP relative to the housekeeping gene ribonuclease P protein subunit p30 (RPP30) was virtually identical for all transfected DNA (Figure 6A). This result establishes that there are no significant differences in transfection efficiencies and that the uracil base excision is not occurring over 24 h. To further establish the retention of uracil in the DNA after transfection of HAP1^ΔUNG^ cells, the levels of uracil in the isolated DNA was compared with the same DNA before transfection using uracil excision qPCR (Ex-qPCR) (17). This method compares the copy number of a DNA isolate that is PCR amplified with and without prior treatment with UNG. Thus, any DNA molecule that contains at least one uracil on each strand is not amplified in the presence of UNG and reduces the copy number compared to the same sample in the absence of UNG. This analysis showed that there was no detectable difference in the uracil content of the transfected DNA compared to the same DNA prior to transfection (Supplemental Figure S3). Thus, the original uracil content was preserved after 24 hours in cells.

We next tested whether dU/A pairs inhibited expression of eGFP mRNA. The CMV-eGFP amplicons were used to transfect HAP1^ΔUNG^ cells for 24h, after which total cellular RNA was extracted, reverse transcribed using random primers to generate the corresponding cDNA and quantified using qPCR with primers directed at the eGFP gene and using glyceraldehyde 3-phosphate dehydrogenase (GAPDH) as a reference. We measured a strong inverse relationship between eGFP mRNA levels and the uracil content of the transfected DNA. For example, following transfection with an amplicon containing only 5% dU/A pairs, a nearly 50% reduction in eGFP mRNA was observed compared to the all T DNA (Figure 6B). The effect of dU/A pairs was even more dramatic for the construct with 75% dU/A pairs, where the eGFP mRNA was less than 1% of the mRNA level obtained with the all T DNA. To evaluate the effect of dU/A pairs on protein expression, the same uracilated amplicons were transfected into HAP1^ΔUNG^ or HAP1^wt^ cells for 24h and eGFP expression was evaluated by flow cytometry (Supplemental Figure S4). The eGFP expressing cells were normalized to the number of eGFP positive cells obtained with the all T DNA. In agreement with the mRNA expression results, a large decrease in the number of eGFP positive cells was observed as the uracil content was increased (Figure 6C). In general, the transfected HAP1^wt^ cells showed a much greater loss in GFP expression at low densities of uracil as compared to the UNG knockout HAP1^ΔUNG^ cells. For example, at only 16% dU/A, nearly all of the eGFP expression was lost in the HAP1^wt^ cells but only a 50% reduction was observed for the HAP1^ΔUNG^ line. This phenomenon likely reflects uracil excision activity of hUNG in HAP1^wt^ cells. The nearly identical eGFP expression levels for the two lines when the DNA contained 5% dU/A, suggested that some of the uracils may be repaired to T/A pairs in HAP1^wt^ cells when their density is low. In contrast, at high uracil density the probability for double strand breaks is high because the frequency of having two closely spaced uracils on different DNA strands is high.

### Transcription fidelity of RNA pol II with uracilated DNA templates in human cells

Given the large inhibitory effects of dU/A base pairs on transcription by RNA pol II, we were interested whether the fidelity of transcription might also be affected. As above with T7 RNAP, we performed EPLD RT-PCR and Sanger DNA sequencing on single clonal cDNAs derived from the mRNA products of transcription from the all T DNA template (eGFP-T) and the template containing 50% dU/A pairs (eGFP-U^50^) templates that were transfected into Hap1^ΔUNG^ cells. In these experiments we used the cellular mRNA that was produced from the partially, rather than fully uracilated template, in order to provide enough RNA for the sequencing goals. Thus, the error frequency for transcribing a completely uracilated DNA template would be larger.

Compared with the <6 × 10^−5^/bp error frequency for RNAs generated from *in vitro* transcription by T7 RNAP, the error frequency for transcripts derived from cellular transcription using the all T template was at least 10-fold greater (6 × 10^−4^/bp) (Table 2). Seven of twenty-one clonal DNAs that were isolated contained single base substitutions of four distinct types. Sixty percent of the substitutions were A→G on the mRNA strand, corresponding to G mispairing with T during transcription. Twenty-eight percent were C→A substitutions on the mRNA strand, corresponding to A mispairing with G on the DNA template, and one T→A mutation was found (14%), which requires pairing of an incoming ATP with A in the template. This spectrum of mutations differs from T7 RNAP in that no insertions were observed. Importantly, it is highly improbable that these mutations arise from the PCR polymerases because the error frequency of 6 × 10^−4^/bp is at least ten times larger than observed for T7 RNAP even though identical PCR reactions were used in both sequencing experiments. This comparatively large substitution frequency arising from cellular transcription by RNA pol II is consistent with previous studies of multi-subunit RNA polymerases (29,30).

**Table 2.** RNA pol II transcription error frequencies arising from DNA templates containing T/A or dU/A pairs

				Transcriptional mutations				
Template^a^	Clones sequenced^b^	Bases sequenced^c^	Mutated clones	Clones with multiple mutations	substitution	deletion	insertion.	Error frequency^d^ (×10^−4^)
eGFP-T	21	12 180	7	0	7	0	0	6
eGFP-U^50^	17	9860	6	3	11	0	0	11

^a^The DNA templates were obtained by PCR amplification using Taq DNA polymerase (see Materials and Methods).

^b^Clonal DNA was obtained by limiting-dilution PCR. Single molecule conditions were assumed when only one of five replicate dilutions gave a positive PCR signal (∼90% probability of being clonal).

^c^Total base pairs sequenced is the sum over all clones sequenced.

^d^Mutation frequency = total mutations/total bases sequenced.

For RNA pol II, the mRNA errors appearing from transcribing the DNA template containing 50% dU/A pairs were not significantly increased as compared to the T template (11 × 10^−4^/bp), Table 2). This result differs from T7 RNAP, where the error frequency was significantly increased using a fully uracilated DNA template (Table 1). Even though the substitution frequencies were similar, RNA pol II transcription of the uracilated DNA template did produce a distinct mutational spectrum compared to the all T template. First, three out of six clones derived from the uracilated template contained multiple mutations, with two to four mutations contained in each clonal sequence. It can be shown that the likelihood of RNA pol II making two or four base substitutions in a single 580 bp transcript is only 6% and 0.1% respectively, as calculated using the Poisson equation and the observed error frequency of 11 × 10^−4^/bp. Thus, the underlying assumption of independent substitution events may not always apply for RNA pol II transcription of templates containing frequent dU/A pairs. We found no obvious reason for the higher than expected number of mutations in these three clones, but none of the mutations were within 25 bp, which makes direct synergistic effects unlikely. The spectrum of substitution mutations was also different for the uracilated template. Although A→G substitutions were also among the most prevalent mutations on the mRNA strand (27%), an equal frequency of G→A substitutions was observed (27%), as well as T→C (18%), C→U (18%), and A→C (9%) substitutions. Possible explanations for this spectrum of substitutions is presented in the Discussion.

## DISCUSSION

### Modified DNA bases and gene regulation

In the last ten years the repertoire of non-canonical DNA bases that are implicated in gene regulation has grown tremendously. These bases now include not only 5-methyl cytosine (5mC), but also its three oxidized forms 5-hydroxymethylcytosine (5hmC), 5-formylcytosine (5fC) and 5-carboxycytosine (5caC) (38,39). Although 5mC serves to silence transcription when present in gene promoters, its enzymatic oxidation by α-ketoglutarate dependent TET enzymes provides a pathway for methylation reversal that ultimately involves base replacement by the base excision repair (BER) pathway (39). An additional example of gene activation involving a modified base and base excision is the ROS-mediated oxidation of DNA to yield 8-oxo-7,8-dihydroguanine (OG) in gene promoters that contain guanine-rich, G-quadruplex–forming sequences (40). In this example, BER by 8-oxoguanine DNA glycosylase (OGG1) yields an abasic site (AP) that promotes a G-quadruplex fold in which apurinic/apyrimidinic endonuclease 1 (APE1) binds and activates gene transcription. It is notable that both of these examples of gene regulation involve the use of components of the BER pathway.

### Uracil as a transcriptional regulator in non-dividing cells

A common theme suggested from the above transcriptional regulation systems is that the outcome is dependent on the BER status of the cell and the location and density of the modified bases. Using a model cell line that lacked any uracil excision activity, we previously reported that uracilated HIV-1 cDNA was integrated efficiently, but the presence of uracil inhibited LTR promoter-initiated transcription of viral genes (7,17). In contrast, during infections of the wild-type line that expressed high levels of hUNG2, the uracilated viral cDNA was efficiently destroyed. These results showed that the fate of uracilated viral DNA was dependent on hUNG2 expression and led to the hypothesis that uracilated proviruses would persist and be transcriptionally down-regulated in non-dividing macrophages because these cells have very low hUNG2 expression (17). This hypothesis was later tested and confirmed in direct infections of human monocyte-derived macrophages (7). Thus, uracil in proviral DNA is strongly implicated as a new mechanism for down-regulating HIV gene transcription in macrophages. These findings also suggest that uracil might also have a global impact on gene transcription under conditions where uracil can persist (i.e. low BER activity) and is not diluted by TTP incorporation during DNA replication (i.e. non-dividing post-mitotic cell types). Two cell types that meet these criteria are long-lived neurons and microglial cells of the brain (41). These ideas suggest that random accumulation of uracil in promoter regions over long periods of time could contribute to pathologies such as neurological and inflammatory diseases.

### Molecular mechanisms based on T7 RNAP promoter structure

**Figure 7.**
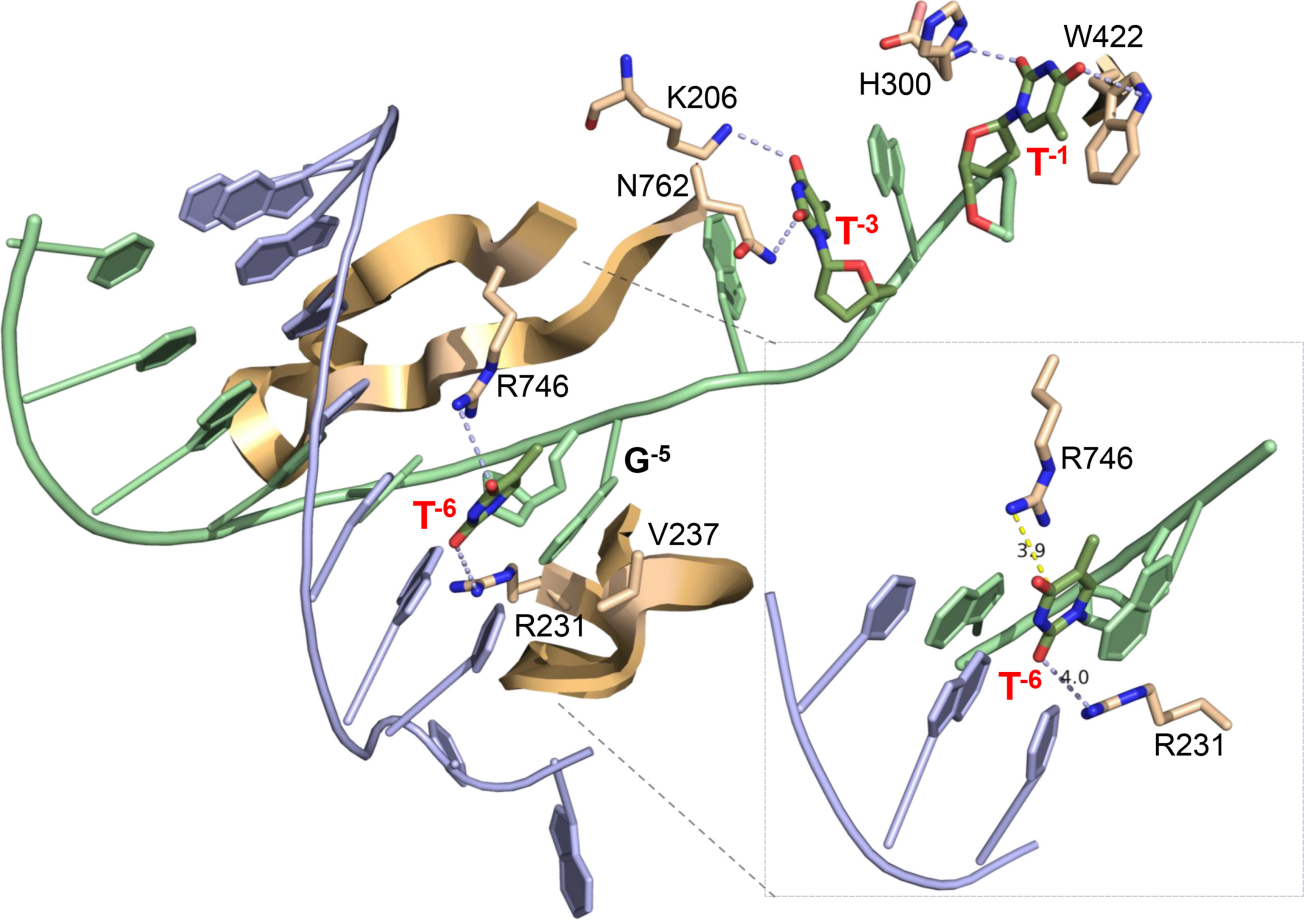
Interactions between T7 promotor DNA and T7RNAP. Formation of the transcriptional bubble begins at the –7 to –5 region of the template strand of DNA, where base pairs –4 through –1 are subsequently melted by T7RNAP and the template strand is directed into the active site of the polymerase. The key thymidine substitutions that produced effects on transcription are indicated (T-6, T-3, T-1, see text). The expanded inset shows the interactions involving T^−6^. PDB accession number 1CEZ.

The relatively simple T7 transcription system provides a useful tool for understanding the potential molecular mechanisms for transcription inhibition arising from uracilation and the crystal structure of T7 RNAP bound to its promoter-initiation DNA sequence provides structural insights (Figure 7) (42). Of particular interest is the molecular basis of the substantial inhibitory effects of substituting T^−6^ with uracil (Figure 7, inset). This structure shows that the transcriptional bubble begins at the –7 to –5 region of the template strand of DNA and that template bases –4 through –1 are completely unpaired. The unpaired strand is held in position by direct interactions with amino acid side chains and is directed into the active site of the polymerase. Of note, base T^−6^ on the template strand shows direct contacts with two positively charged protein side chains, Arg231 and Arg746, which are located on the specificity and β-hairpin loops, respectively. Arg231 is centered over the face of T^−6^ forming a pi-cation interaction with the pyrimidine ring (3.7 Å), while the side chain of Arg746 points directly at the 4-keto group of T^−6^ forming a potential hydrogen bond or electrostatic interaction. In addition, T^−6^ is paired with the non-template adenine and stacked with G^−5^ and G^−7^ on the template strand (Figure 7, inset).

These interactions suggest that the large inhibitory effect of the T^−6^→U^−6^ substitution arises from disruption of the nucleic acid structure in this region. This type of mechanism is consistent with the known energetic destabilization arising from T/A→dU/A substitutions. Uracil-induced destabilization is manifested as increased dU/A base pair dynamics and decreased thermal and enthalpic stability of duplexes containing dU/A pairs (43–45). The increased dynamics of dU/A pairs has been studied using the NMR imino proton exchange method and attributed to the reduced electron density of the base, which leads to poorer π stacking interactions with neighboring bases (44). Additionally, the more electron dense thymidine base would be expected to form a stronger pi-cation interaction with Arg231. We propose that uracil-induced destabilization of the junction between single strand and duplex DNA at the transcription bubble has a long-range effect that propagates to the polymerase active site and perturbs positioning of the template.

Another key aspect of our findings is the observation that substitutions at T^−1^ and T^−3^ restore the activity that was lost with the T^−6^ substitution (Figure 3). This aspect of the data is difficult to confidently rationalize based on a static crystal structure, but long-range compensatory effects on accurate strand positioning are also suggested. Both T^−1^ and T^−3^ are located in the unpaired template strand that binds to the enzyme. Residue T^−1^ is notably stacked with a tryptophan residue that is responsible for positioning the template for initiation and T^−3^ is stacked with neighboring G^−2^ and G^−4^ (42). Thus, as suggested above for T^−6^, uracil-induced disruption of aromatic stacking interactions could ‘rescue’ the effect of the T^−6^ substitution through long-range compensatory effects that promote primer extension.

### dU/A pairs and T7 RNAP error frequencies

**Figure 8.**
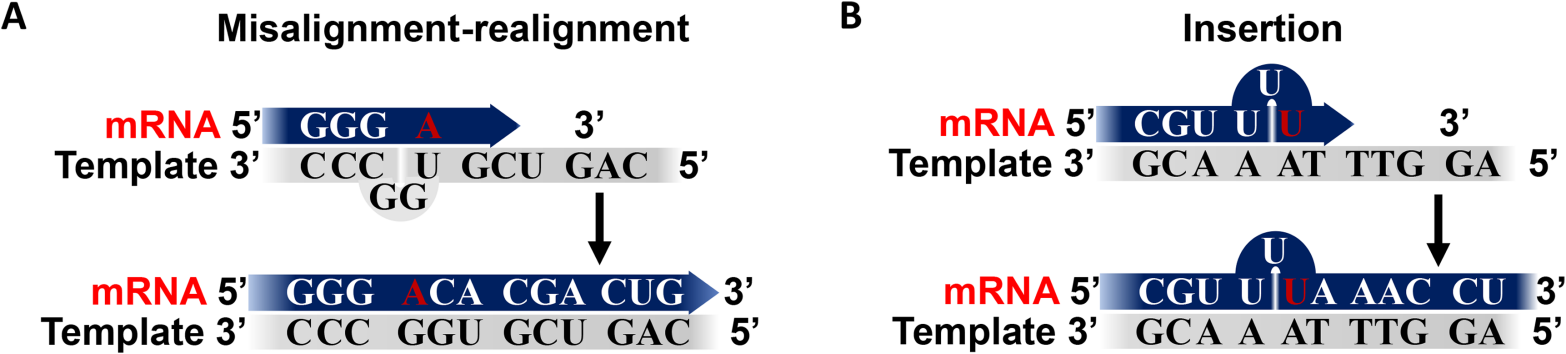
Possible mutational mechanisms with T7 RNAP arising from uracilated DNA templates. (**A**) Substitutions by misalignment. (**B**) Insertion mutation by slippage of the extending mRNA.

We observed a C→A transversion and a single U insertion in the sequencing of mRNA transcripts derived from a uracilated DNA template and no mutations in 19 single molecule isolates and 18,000 bp of sequence derived from an all T/A template (Table 1, Figure 5). Notably, neither of these mutations occurred at positions where uracil would have been present on the template strand and suggests uracilated DNA templates have an indirect effect on transcription fidelity rather than a mechanism that involves greater mispairing between incoming NTPs and dU on the template strand. In principle, a C→A transversion could arise from ATP mis-pairing with dG on the template strand or through a misalignment mechanism involving pairing with an n^+1^ T or U base on the template strand (Figure 8A). A standard misalignment mechanism is not possible because the sequence context of the substitution shows that the n^+1^ base is dG. However, a misalignment mechanism could be followed if two dG bases on the template strand flipped such that the ATP could pair with the n^+2^ dU (Figure 8A). Such a mechanism might be promoted by the duplex destabilizing properties of uracil, but its plausibility is not known at this time. A mispairing mechanism between ATP and a template dG is unfavorable due to steric clashes, and accordingly, ATP shows very weak binding affinity for a template dG bound to T7 RNAP and the frequency of C→A transversions is low *in vitro* (1.5 × 10^−7^) (26). Although the error frequency suggested from our limited sequencing data is 1000-fold greater (1.4 × 10^−4^), the frequencies are dependent on sequence context and C→A transversions are some of the most frequent mutations observed in cellular mRNAs (26,29). The single uracil insertion mutation is readily explained by the sequence context of the insertion (Figure 8B). This insertion occurs after a run of three dA residues on the template strand. Homopolymer runs are known to promote ‘strand slippage’ of the extending strand and lead to insertions (46), in this case by pairing of rUTP with a neighboring dA of the homopolymer run. The propensity for slippage would be expected to increase in the context of a destabilized uracilated DNA template.

### Uracilated DNA templates inhibit transcription by RNA pol II

The single and multiple site uracil substitutions with T7 RNAP are informative because they establish that the presence of even a single uracil can induce large changes in transcriptional activity even in the absence of direct protein interactions. In the context of the more complex transcriptional requirements of human RNA pol II, this suggests that both direct and indirect effects arising from single or multiple uracils are possible. The implication of indirect effects is that such effects could impact binding of transcription factors, regardless of whether they bind to consensus sites that have the potential for T→dU substitutions. Uracils could also directly impact the activity of RNA pol II at the promoter binding, initiation or elongation stages of transcription. Finally, the presence and abundance of uracil in DNA could in principle disrupt or stabilize histone-DNA interactions, which could indirectly affect transcriptional activity. Thus, the net effect of uracilation at a given site would be expected to be context dependent, much like the combinatorial effects of other epigenetic modifications. In this study, we have observed complete inhibition of transcription from a highly uracilated CMV promoter in UBER-deficient human cells (Figure 6). This profound effect, which exceeds that of the T7 RNAP system, strongly suggests that uracil is having a pleotropic effect on multiple aspects of transcription as suggested above. A further understanding of these effects will require experimental designs that allow the correlation of specific uracil positions in gene promoters with transcription output. Ideally, these studies will ultimately be done in the context of human genomic DNA.

### RNA base substitutions from RNA pol II transcription in cells

**Figure 9.**
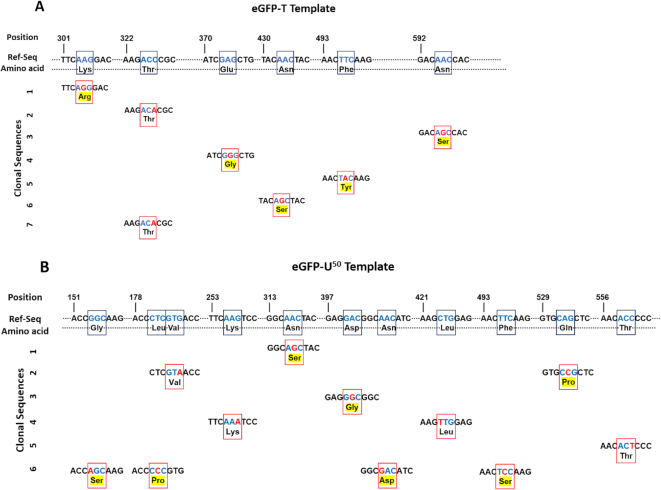
Mutational spectrum of human RNA pol II using all T and partially uracilated transcriptional templates and effects on protein sequence. **(A)** Mutation analysis of RNA pol II on eGFP-T template. There are seven clones containing seven mutations, no multiple mutations observed in any of the clones. (**B**) Mutation analysis of RNA pol II on eGFP-U^50^ template. There are six clones containing eleven mutations, three clones contain multiple mutations. Black boxes refer to the codon and corresponding amino acid of the reference sequence. Red boxes refer to the codon and corresponding amino acid of the clonal sequences. Blue letters refer to the codon where mutations occur. Red letters refer to the position of the substitution and the numbers on the top of the sequence correspond to the positions on the original pCMV-GFP plasmid sequence. Amino acid mutations are highlighted with yellow color. These mutations were observed on both strands of the Sanger sequencing reads.

We found much higher rates of base substitutions during transcription by RNA pol II in Hap1^ΔUNG^ cells when the template contained all T or 50% dU. For both templates, the largest fraction of substitutions involved G/T or G/dU wobble mispairs during transcription (GTP incorporated opposite to T or dU in the template), which are frequently observed polymerase errors and previously reported for cellular transcription by RNA pol II (47,48). In this data, there was no evidence that the presence of dU in the template increased the frequency of such wobble mispairing. In many cases, these substitutions led to changes in the amino acid sequence (Figure 9). With the all T template, two C→A transversions at the same site were observed in two separate clonal isolates (Figure 9A). It is highly unlikely that these identical sequences arise from contamination during EPLD-PCR, because these clonal DNA samples were never in close proximity. Thus, this sequence context may represent a hot spot for incorporation of ATP opposite to G on the DNA template. Another clonal isolate from the all T sample contained a T→A transversion (Figure 9A), which would require ATP mispairing with A on the template. The mechanistic basis for these two types of purine-purine mispairings with incoming ATP is not obvious from the sequence context, but these events are independent of the uracil status of the template and have been observed previously for cellular transcription by RNA pol II (49).

In addition to A→G and C→U transitions involving wobble base pairs, which comprised 45% of the observed substitutions, several unique base substitutions were observed with the uracilated DNA template that resulted in codon changes (Figure 9B). Many of these substitutions can be rationalized in terms of a misalignment/realignment mechanism that could be promoted by the reduced stability of rA/dU pairs in the paired nascent RNA/DNA hybrid. Five such substitutions involving G→A and T→C transitions on the mRNA strand are observed and reside in sequence contexts that could promote a misalignment/realignment mechanism for base substitution (Figure 9B). The implication of these findings is that uracilated DNA templates could increase the diversity of protein sequences that are translated. Given the frequency of substitution mutations of ∼10^−3^/bp and the assumption that only one in three substitutions gives rise to a change in the amino acid sequence, a protein with 1000 amino acids would on average contain one amino acid change, and 37% of the protein sequences of this length would have zero changes. This frequency of mRNA sequence changes resulting from transcription of a linear DNA template is comparable to previous whole genome studies that have compared RNA transcript sequences with the corresponding genomic DNA template (49). Thus, there appears to be a surprisingly low fidelity requirement for human RNA pol II.

## DATA AVAILABILITY

The authors declare that the data supporting the findings of this study are contained with the paper and its supplementary information files. Raw data files are available from the corresponding author upon reasonable request.

## Supplementary Material

Supplementary Data
