# Supplementary material for: Deoxyuridine in DNA has an inhibitory and promutagenic effect on RNA transcription by diverse RNA polymerases
Source: Nucleic Acids Res. 2019 Mar 20;47(8):4153–68. doi: 10.1093/nar/gkz183 (PMC6486633; doi:10.1093/nar/gkz183)
Supplement: Supplementary Data [file gkz183_supplemental_files.pdf]

Supporting information for:

Deoxyuridine in DNA has an Inhibitory and Promutagenic Effect on RNA Transcription by Diverse RNA Polymerases

Junru Cui, Anthony Gizzi, James T. Stivers*

Department of Pharmacology and Molecular Sciences, The Johns Hopkins University School of Medicine, 725 North Wolfe Street, Baltimore, MD 21205-2185, USA

*To whom correspondence should be addressed. E-mail: jstivers@jhmi.edu. Phone: (410) 502-2758.

DNA Sequences

S38 sequences

T38

5′-TTA ATA CGA CTC ACT ATA GGG GAA TTG TGA GCG GAT AAC-3′

3′-AAT TAT GCT GAG TGA TAT CCC CTT AAC ACT CGC CTA TTG-5′

$U^{I}38$

5′-TTA ATA CGA CTC ACT ATA GGG GAA TTG TGA GCG GAT AAC-3′

3′-AAT TAT GCT GAG TGA TAT CCC CUU AAC ACT CGC CTA TTG-5′

$U^{IE}38$

5′-TTA ATA CGA CTC ACT ATA GGG GAA TTG TGA GCG GAT AAC-3′

3′-AAT TAT GCT GAG TGA TAT CCC CUU AAC ACU CGC CUA UUG-5′

$U^{PIE}38$

5′-TTA ATA CGA CTC ACT ATA GGG GAA TTG TGA GCG GAT AAC-3′

3′-AAU UAU GCU GAG UGA UAU CCC CUU AAC ACU CGC CUA UUG-5′

S321 sequences

Amplicon sequence 119-440 of pET30a plasmid

Non-template strand

5′-
GAAATTAATACGACTCACTATAGGGGAATTGTGAGCGGATAACAATTCCCCTCTAGAAATAATT
TTGTTTAACTTTAAGAAGGAGATATACATATGCACCATCATCATCATCATTCTTCTGGTCTGGT
GCCACGCGGTTCTGGTATGAAAGAAACCGCTGCTGCTAAATTCGAACGCCAGCACATGGACAGC
CCAGATCTGGGTACCGACGACGACGACAAGGCCATGGCTGATATCGGATCCGAATTCGAGCTCC
GTCGACAAGCTTGCGGCCGCACTCGAGCACCACCACCACCACCACTGAGATCCGGCTGCTAACA
AA-3′

Template strand

3′-
CTTTAATTATGCTGAGTGATATCCCCTTAACACTCGCCTATTGTTAAGGGGAGATCTTTATTAA
AACAAATTGAAATTCTTCCTCTATATGTATACGTGGTAGTAGTAGTAGTAAGAAGACCAGACCA
CGGTGCGCCAAGACCATACTTTCTTTGGCGACGACGATTTAAGCTTGCGGTCGTGTACCTGTCG
GGTCTAGACCCATGGCTGCTGCTGCTGTTCCGGTACCGACTATAGCCTAGGCTTAAGCTCGAGG
CAGCTGTTCGAACGCCGGCGTGAGCTCGTGGTGGTGGTGGTGGTGACTCTAGGCCGACGATTGT
TT-5′

S23 Sequences

See **Table S2**

Table S1. Transcription kinetics using DNA substrate S321 with random dU/A pairs

Substrate[a]	k_{cat} (min^{-1})	K_m (nM)	k_{cat}/K_m	k_{cat}/K_m^{rel} [b]
T321	290 ± 19	14 ± 3	20 ± 4	1.0 ± 0.3
U^{50}321	90 ± 7	10 ± 3	9 ± 3	0.46 ± 0.2
U321	89 ± 21	184.2 ± 64	0.5 ± 0.2	0.024 ± 0.01

[a]Substrates (321 bp) were prepared containing just T/A (T321) or 50 and 100% dU/A base pairs (U^{50}321 and U321). [b]k_{cat}/K_m^{rel} is the relative catalytic efficiency calculated using the ratio $(k_{cat}/K_m)^{U\ promoter}/(k_{cat}/K_m)^{T\ promoter}$. The concentrations of DNA in each reaction were 1-60 nM for T321 and U^{50}321, and 5-120 nM for U321. The concentration of T7 RNAP was 5 nM. The concentration of dNTPs was 0.5 mM each and α-^{32}P-GTP was 100 μCi/mL. The reactions were incubated at 37°C.

Table S2. Transcription initiation kinetics from S23 uracilated promoters[a]

Substrate[b]	Promoter sequences	k_{cat} (min^{-1})	K_m (nM)	k_{cat}/K_m^{rel} [c]
	-6 -3 -1 +1 +3 +5			
**T23**	CTA ATA CGA CTC ACT ATA GGA CT<br>GAT TAT GCT GAG TGA TAT **CCT GA**	141.5 ± 10.4	30.5 ± 6.2	1 ± 0.2
**U^{nt}23**	C**U**A A**U**A CGA C**U**C AC**U** A**U**A GGA C**U**<br>GAT TAT GCT GAG TGA TAT **CCT GA**	135.7 ± 14.3	60.1 ± 13.3	0.5 ± 0.1
**U^{t}23**	CTA ATA CGA CTC ACT ATA GGA CT<br>GA**U** **U**A**U** GC**U** GAG **U**GA **U**A**U** **CCU GA**	83.2 ± 34.1	259.1 ± 143.4	0.06 ± 0.04
**U23**	C**U**A A**U**A CGA C**U**C AC**U** A**U**A GGA C**U**<br>GA**U** **U**A**U** GC**U** GAG **U**GA **U**A**U** **CCU GA**	20.6 ± 6.7	207.6 ± 96.1	0.02 ± 0.01
**U^{-1}23**	CTA ATA CGA CTC ACT ATA GGA CT<br>GAT TAT GCT GAG TGA TA**U** **CCT GA**	133.1 ± 31.8	65.3 ± 32.6	0.4 ± 0.2
**U^{-3}23**	CTA ATA CGA CTC ACT ATA GGA CT<br>GAT TAT GCT GAG TGA **U**AT **CCT GA**	137.4 ± 20.8	26.7 ± 12.4	1.1 ± 0.5
**U^{-6}23**	CTA ATA CGA CTC ACT ATA GGA CT<br>GAT TAT GCT GAG **U**GA TAT **CCT GA**	34.7 ± 2.7	141.7 ± 25.5	0.04 ± 0.02
**U^{+3}23**	CTA ATA CGA CTC ACT ATA GGA CT<br>GAT TAT GCT GAG TGA TAT **CCU GA**	88.8 ± 12.4	58.7 ± 17.9	0.3 ± 0.06
**U$^{+3,-6}$23**	CTA ATA CGA CTC ACT ATA GGA CT<br>GAT TAT GCT GAG **U**GA TAT **CCU GA**	90.4 ± 11.0	101.2 ± 22.0	0.3 ± 0.08
**U$^{-1,-6}$23**	CTA ATA CGA CTC ACT ATA GGA CT<br>GAT TAT GCT GAG **U**GA TA**U** **CCT GA**	108.5 ± 7.8	132.2 ± 22.2	0.2 ± 0.06
**U$^{-3,-6}$23**	CTA ATA CGA CTC ACT ATA GGA CT<br>GAT TAT GCT GAG **U**GA **U**AT **CCT GA**	167.2 ± 6.9	188.2 ± 16.1	0.2 ± 0.08
**U$^{-1,-3,-6}$23**	CTA ATA CGA CTC ACT ATA GGA CT<br>GAT TAT GCT GAG **U**GA **U**A**U** **CCT GA**	143.9 ± 5.1	146.8 ± 11.7	0.2 ± 0.09

[a]The superscripts nt and t refer to the non-template and template strands, respectively. The numbering -1, -3, -6 refers to position of the T→U substitution relative to the transcription start site (+1). [b]The number 23 refers to the length of the substrate. [c]k_{cat}/K_m^{rel} is the relative catalytic efficiency calculated using the ratio $(k_{cat}/K_m)^{\text{uracilated promoter}}/(k_{cat}/K_m)^{\text{T promoter}}$ The concentration of DNA in each reaction used as 5-120 nM and that of T7 RNAP was 10 nM. The concentration of dNTPs was 0.5 mM each and that of α-^{32}P-GTP was 100 µCi/mL. The reactions were incubated at 37°C. Five µL portions of the reaction were removed at 0, 5, and 10 min and mixed with 15 µL of loading buffer (98% formamide, 10 mM EDTA). The transcription products were separated using denaturing PAGE (8 M urea, 1× TBE, 14% acrylamide).

Table S3. Transcription Kinetics from PIE duplexes

Substrate[a]	k_{cat} (min^{-1})	K_m (nM)	k_{cat}/K_m	$k_{cat}/K_m^{rel\ b}$
T38	12 ± 2	530 ± 170	0.02 ± 0.008	1.0 ± 0.5
$U^{I}38$	10 ± 2	740 ± 200	0.01 ± 0.003	0.6 ± 0.3
$U^{IE}38$	2.6 ± 0.4	340 ± 120	0.008 ± 0.003	0.3 ± 0.1
$U^{PIE}38$	1.9 ± 0.2	465 ± 80	0.004 ± 0.0008	0.2 ± 0.05

[a]The superscripts I, IE and PIE refer to the region containing initiation, initiation and elongation, promoter, initiation and elongation, respectively. The number 38 refers to the length of the substrate. [b]k_{cat}/K_m^{rel} is the relative catalytic efficiency calculated using the ratio $(k_{cat}/K_m)^{\text{uracilated substrate}}/(k_{cat}/K_m)^{\text{T substrate.}}$ The concentration of DNA in each reaction used as 50-600 nM and that of T7 RNAP was 20 nM. The concentration of dNTPs was 0.5 mM each and that of α-^{32}P-GTP was 100 µCi/mL. The reactions were incubated at 37°C. Five µL portions of the reaction were removed at 0, 4, and 8 min and mixed with 15 µL of loading buffer (98% formamide, 10 mM EDTA). The transcription products were separated using denaturing PAGE (8 M urea, 1× TBE, 14% acrylamide).

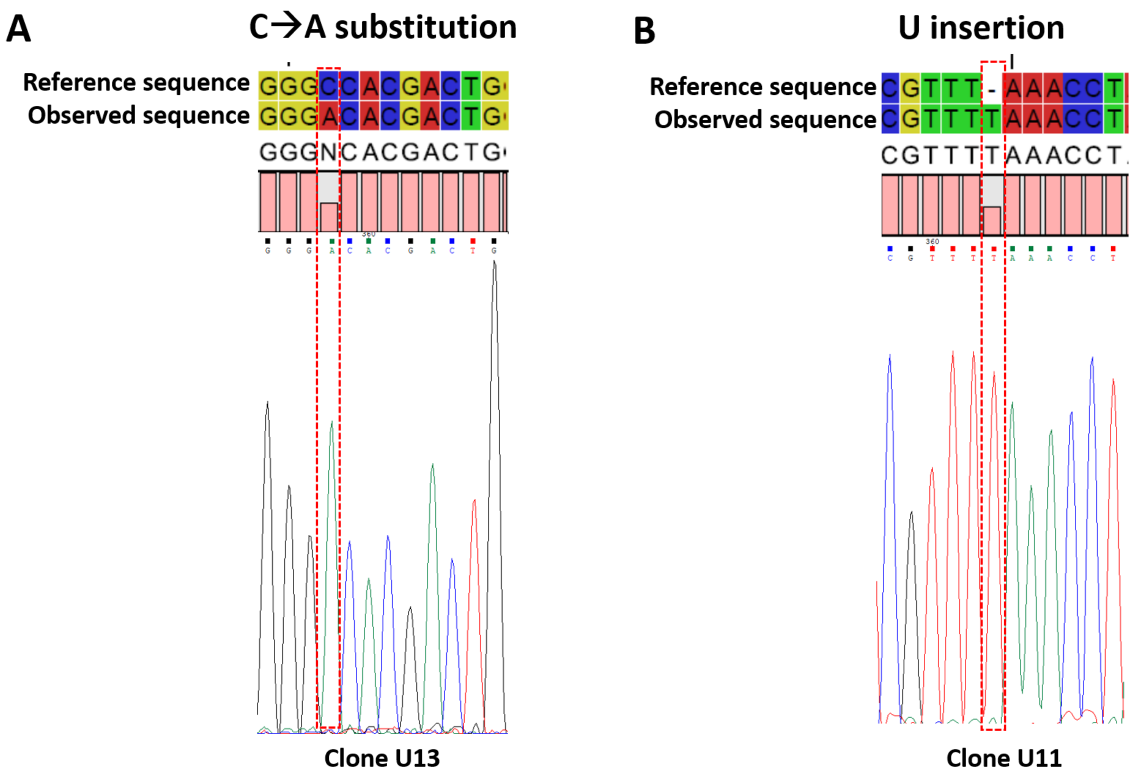

Figure S1: Sequence alignment and trace map showing mutations of U1095 DNA template. Sequence alignment generated using CLC Sequence Viewer 8.0 where the reference sequence is on the top. Fwd-sequence is the reverse complementary sequence of the original sequence obtained using the FWD primer for alignment purpose only. U13, U11 are the two clones showing mutations. R and F refer to trace file associated to the REV primer and FWD primer respectively. Red dash line indicates where the corresponding peak for the mutation. U11-Fs refers to the sequences obtained using a primer (5'-ACTGGAGCCTGAGGAGTT-3') targeting a fragment of U1095 to confirm the insertion.

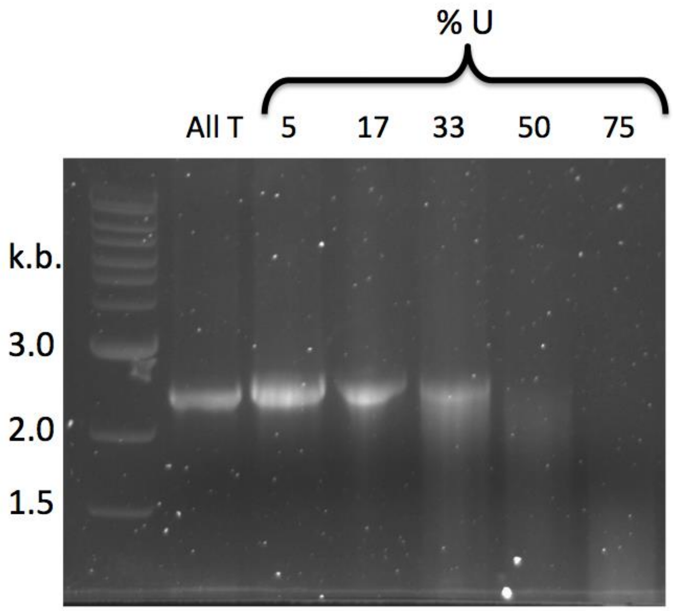

Figure S2: To confirm the presence of uracil in DNA obtained from PCR amplification in the presence of various ratios dUTP/[dUTP + TTP], each linear amplicon was digested with UNG before analysis by agarose gel electrophoresis (1%). DNA fragmentation by UNG increases with the abundance of U/A pairs in the DNA. The percentage of dUTP used in the amplification is indicated above each lane.

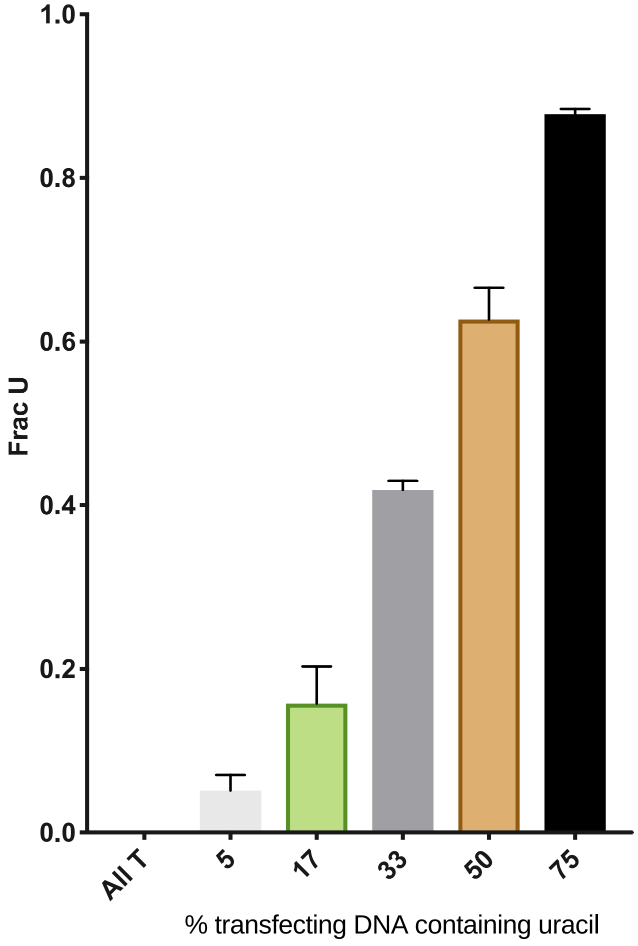

Figure S3. Uracils are retained in transfected DNA for 24 hours. To confirm that DNA uracils were retained after transfection into HAP1$^{\Delta UNG}$ cells that are defective in excision of uracil from U/A base pairs, the cells were lysed 24 hours after transfection and the DNA was isolated and analyzed by uracil excision qPCR (Ex-qPCR)(see Methods). The fraction of the eGFP amplicons that contain uracil (FracU) was calculated from the Ct values using eq 3 (Materials and Methods). The data confirms that the original U/A pairs persisted in the UBER deficient cells for 24 hours.

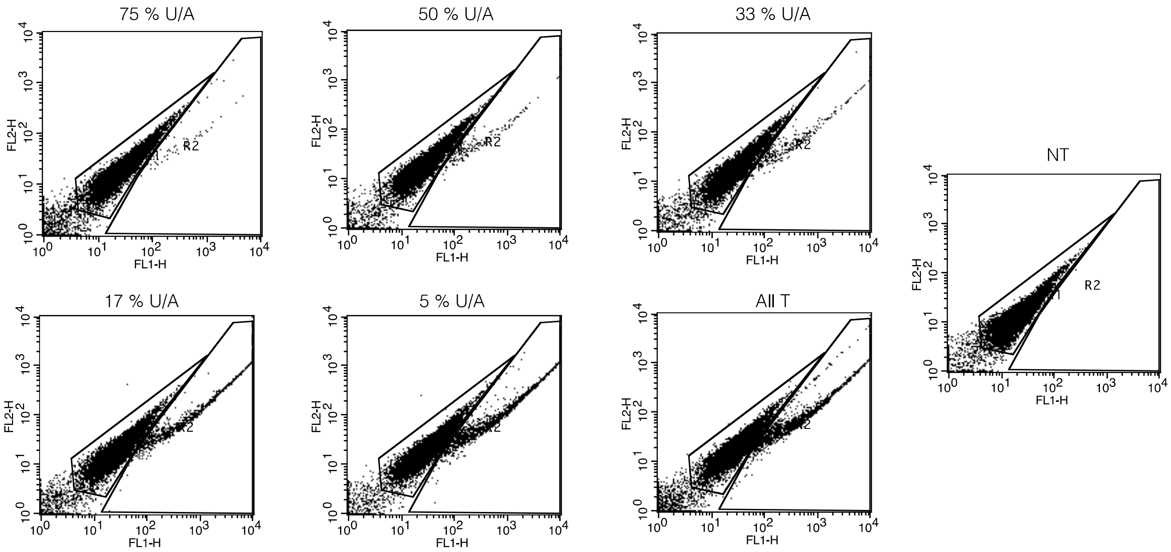

Figure S4. **The number of eGFP expressing HAP1$^{\Delta UNG}$ cells is inversely correlated with the abundance of U/A pairs in the transfected DNA.** HAP1$^{\Delta UNG}$ cells were transfected with ~2500 bp linear DNAs with increasing levels of U/A pairs (0-75%) and containing an eGFP expression cassette. eGFP expression was measured using flow cytometry 24 hours after transfection. The percentage of eGFP positive cells is defined as (% eGFP positive cells for the U-DNA transfection)/(% eGFP positive cells for the all T-DNA transfection). The cells were gated on size and granularity (FSC and SSC) to obtain a homogeneous cell population, and then by eGFP intensity (shown as R2 above). The FL1-H channel (x-axis) is eGFP intensity (488 nm excitation, 530 nm emission) and the FL2-H channel (y-axis) is an orange fluorescence negative control (488 nm excitation, 585 nm emission). The percentage of dUTP used during the PCR synthesis of the DNA expression constructs is defined as 100 x [dUTP]/[TTP + dUTP]. The no transfection (NT) control is shown on the right.
